# *WLP3* Encodes the Ribosomal Protein L18 and Regulates Chloroplast Development in Rice

**DOI:** 10.1186/s12284-023-00674-9

**Published:** 2023-12-13

**Authors:** Tao Lu, Wenjin Yin, Yinuo Zhang, Chaoyu Zhu, Qianqian Zhong, Sanfeng Li, Nuo Wang, Zhengai Chen, Hanfei Ye, Yuan Fang, Dan Mu, Yuexing Wang, Yuchun Rao

**Affiliations:** 1 College of Life Sciences, Zhejiang Normal University, Jinhua, 321000 Zhejiang China; 2 State Key Laboratory of Rice Biology and Breeding, China National Rice Research Institute, Hangzhou, 310006 China; 3 College of Life Sciences, Anqing Normal University, Anqing, 246133 Anhui China

**Keywords:** Rice, Ribosomal proteins, White leaf and panicle, Chloroplast development, Abiotic stresses

## Abstract

Plastid ribosomal proteins play a crucial role in the growth and development of plants, mainly in the gene expression and translation of key genes in chloroplasts. While some information is known about the regulatory processes of plastid ribosomal proteins in various plant species, there is limited knowledge about the underlying mechanisms in rice. In this study, ethyl methanesulfonate (EMS) mutagenesis was used to generate a new mutant called *wlp3* (*white leaf and panicle3*), characterized by white or albino leaves and panicles, which exhibited this phenotype from the second leaf stage until tillering. Furthermore, after a certain period, the newly emerging leaves developed the same phenotype as the rice variety ZH11, while the albino leaves of *wlp3* showed an incomplete chloroplast structure and significantly low chlorophyll content. A transition mutation (T to C) at position 380 was identified in the coding region of the *LOC_Os03g61260* gene, resulting in the substitution of isoleucine by threonine during translation. *WLP3* encodes the ribosomal L18 subunit, which is localized in the chloroplast. Complementation experiments confirmed that *LOC_Os03g61260* was responsible for the albino phenotype in rice. *WLP3* has high expression in the coleoptile, leaves at the three-leaf stage, and panicles at the heading stage. Compared to the wild-type (WT), *wlp3* exhibited reduced chlorophyll synthesis and significantly decreased expression levels of genes associated with plastid development. Yeast two-hybrid (Y2H) analysis revealed that WLP3 interacts with other ribosomal subunits, to influence chloroplast development. These results contribute to a better understanding of the underlying molecular mechanisms of chloroplast development and plastid gene translation.

**Supplementary Information:**

The online version contains supplementary material available at 10.1186/s12284-023-00674-9.

## Background

Chloroplasts are typically 6 µm in length and 3 µm in width and are the key organelles responsible for photosynthesis in higher plants. Mesophyll cells typically house 100–150 chloroplasts, with variations in quantity among different species and organs (Arsova et al. 2010). Chloroplasts are structually compsoed of three main components: the chloroplast membrane, thylakoids, and the stroma. The chloroplast membrane is composed of two layers, including a highly permeable outer layer and an inner layer that demonstrares strong selectivity (Bohrer et al. 2012). Thylakoids are flattened structures consisting of a single layer of membrane which houses numerous photosynthetic pigments and proteins involved in the electron transport system, including cytochrome complex, plastoquinone, plastocyanin, ferredoxin, and flavoprotein. Water can undergo photolysis within the thylakoid membrane to producing H and O_2_, while simultaneously converting ADP (adenosine diphosphate) into ATP (adenosine triphosphate) to supply energy for subsequent dark reactions. Due to its role in photosynthesis, the thylakoid membrane is often referred to as the photosynthetic membrane (Braun et al., 2012). Multiple thylakoids stacked together form gtana structures. The area between the thylakoid and the chloroplast membrane is called the stroma, which contributes significantly to carbon assimilation. The stroma is home to enzymes that are engaged in assimilation as well as the production of chloroplast DNA, protein starch, and other substances. The primary function of the stroma is to fix CO_2_ into organic matter and utilize ATP to convert the three-carbon sugars via the process of reduction, thereby providing ample raw materials for photosynthetic product synthesis (e.g., carbohydrates).

The size of the chloroplast ribosome is 70S, which consists of a large 50S subunit and a 30S small subunit(Barakat et al. 2001). Homologous genes are found in bacteria (Yamaguchi et al., 2000). The 50S large subunit in the chloroplast contains 33 types of ribosomes with 25 being encoded by nuclear genes and transported into the chloroplast via signal peptides. The 30S small subunit features of 25 proteins, 13 of which are encoded by nuclear genes (Yamaguchi et al., 2001). Ribosomal proteins have been extensively studied in *Escherichia coli* (*E. coli*) (Dresios et al., 2001), however, there is comparatively limited research on these proteins in higher plants. The first plastid ribosome protein (PRP) mutant was identified in maize (*hcf60*) exhibiting a pale green phenotype(Vladimirov et al. 2000). This was elucidated by cloning and is caused by a mutation in a gene encoding the small ribosomal subunit proteins (*PRPs17*) (Schultes et al. 2000). In recent years, several rice *PRP* genes have also been cloned including *WGL2* (*PRPS9*) (Qiu et al. 2018), *ASL4* (*PRPS1*) (Zhou et al. 2021), *WLP1* (*PRPL13*) (Song et al. 2014), *ASL2* (*PRPL21*) (Lin et al. 2015), *AL1* (*PRPL12*) (Gong et al. 2013), and *ASL1* (*PRPS20*) (Gong et al. 2013). While ribosomal protein mutations can lead to irreversible effects on translation, certain genes are dispensable for plant growth. The chlorophyll content and photosynthetic rate of the mutants are unaffected enabling the completion of the entire life cycle (Romani et al. 2012). Additionally, some mutants including *al1* and *asl2* display a total albino phenotype during the seedling stage causing the plant to die before the three-leaf stage and therefore not complete the entire life cycle. Other PRP proteins may only play an essential role in some extreme environments. Instance, *wlp1* exhibits a reduction in chlorophyll content at low temperatures. However, *wlp1* content is not significantly different from WT at high temperatures, suggesting that *wlp1* in a low-temperature environment plays a role in maintaining chloroplast stability. In this study, a novel rice mutant *white leaf white panicle 3* (*wlp3*) was isolated. The mutant exhibited an albino phenotype at the seedling stage and recovered green leaves at later developmental stages. Map-based cloning revealed that *WLP3* encodes a large ribosomal subunit L18. The results have demonstrated that *WLP3* plays an important role in the early developmental stages of rice chloroplasts.

## Results

### *wlp3* Albino Phenotype at the Seedling Stage

**Fig. 1:**
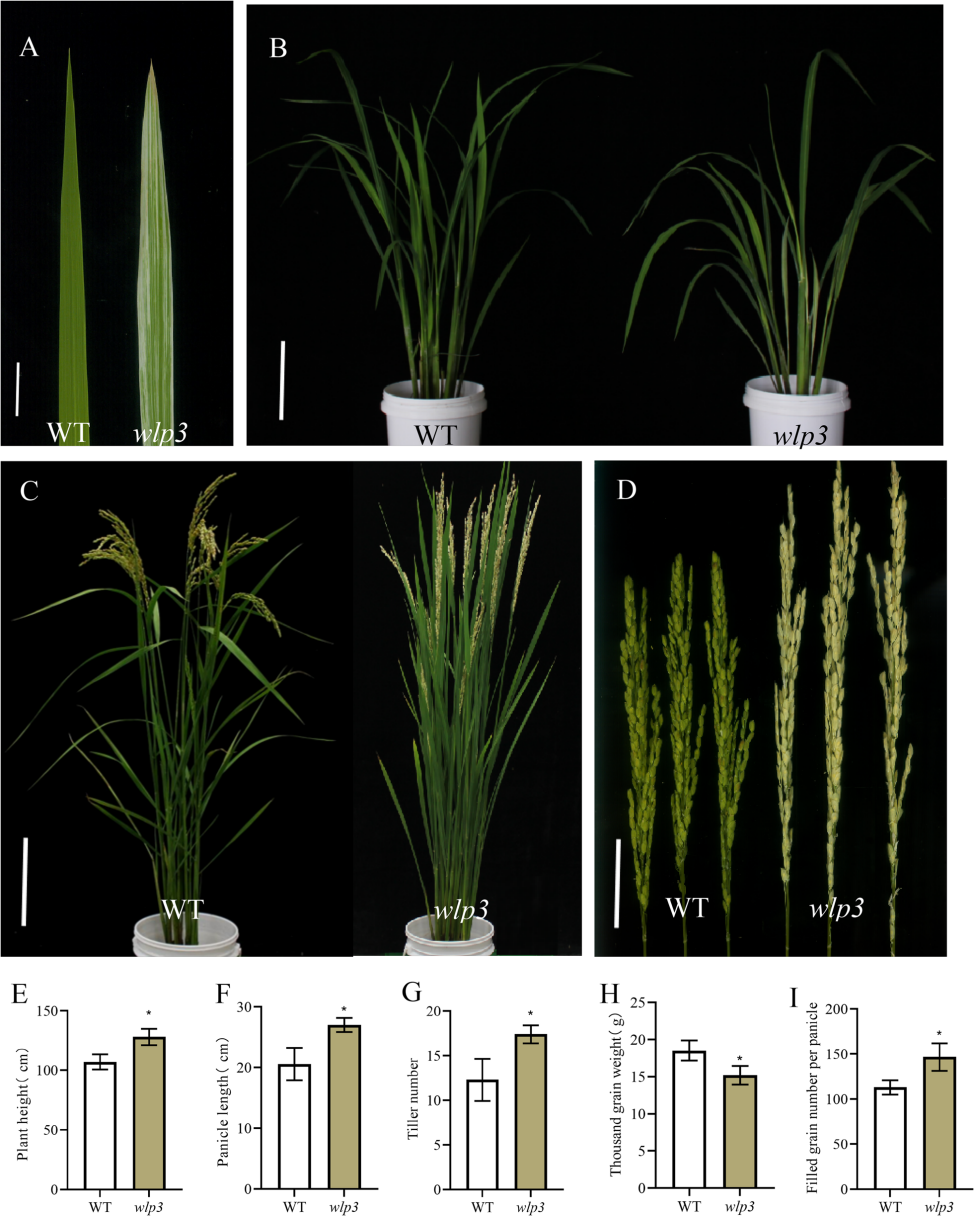
WT and *wlp3* phenotypes. **A** Leaves at the seedling stage (scale bar = 1 cm). **B** The tillering stage (scale bar = 10 cm). **C** Map at the mature stage (scale bar = 20 cm). **D** Spike phenotype maps (scale bar = 5 cm). **E** Plant height*.* **F** Panicle length*.* **G** Tiller number*.* **H** Thousand-grain weight*.* **I** Filled grain number per panicle

**Fig. 2:**
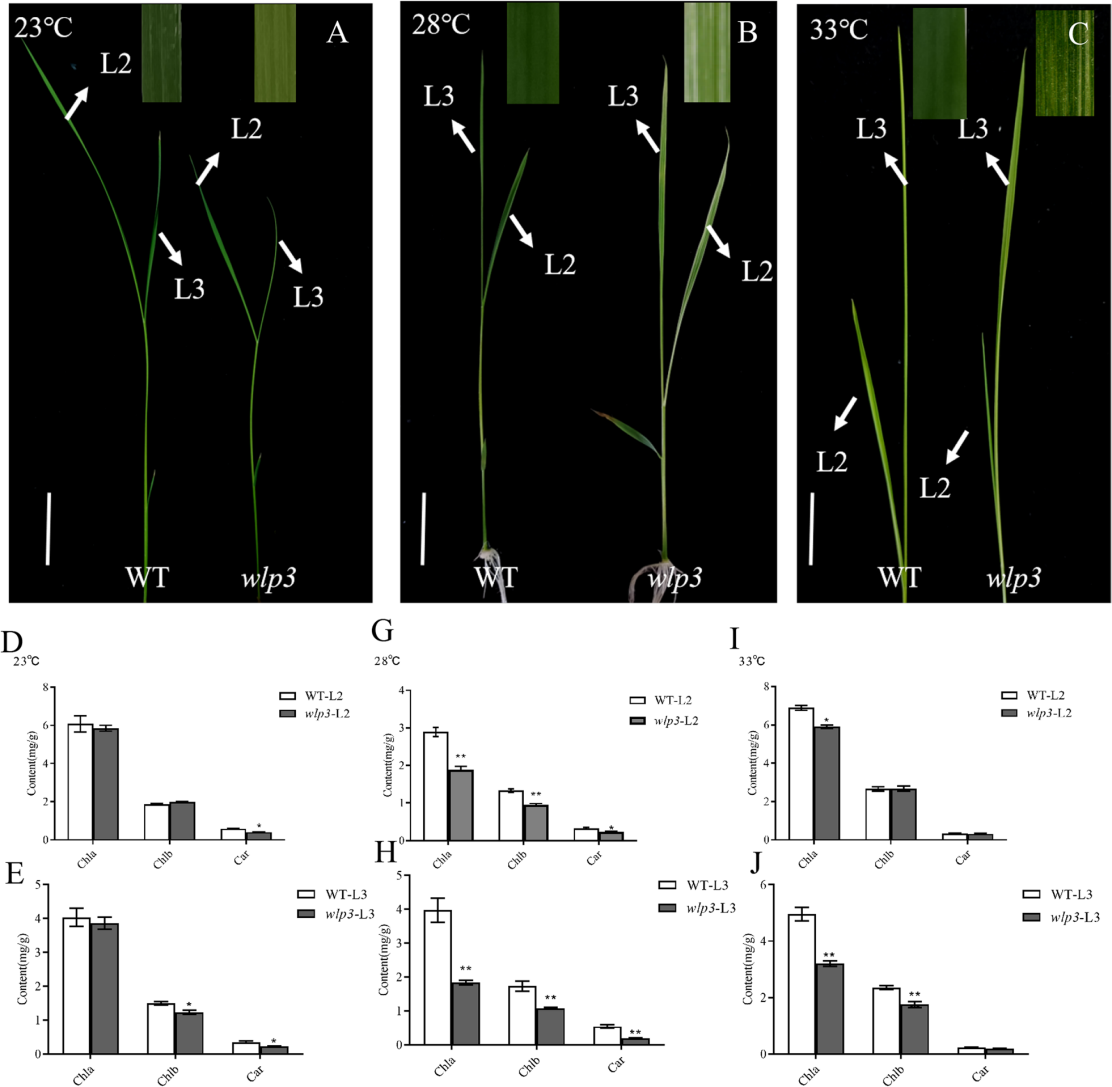
Phenotype of WT and *wlp3* leaves and chlorophyll content of second and third leaves at different temperatures. **A**–**C** Leaf phenotype maps at 23, 28, and 33 °C (scale bar = 4 cm). **D**–**H** Chlorophyll content of the second and third leaves treated at 23 °C. **G**–**H** Chlorophyll content of the second and third leaves treated at 28 °C. **I**–**J** Chlorophyll content of the second and third leaves treated at 33 °C

Under field conditions, the *wlp3* mutant exhibited a white stripe phenotype at the two-leaf stage that persisted through the tillering stage (Fig. 1A and B). The white stripes were distributed along the leaf veins of the entire leaf. After the four-leaf stage, the newly emerging *wlp3* leaves gradually developed a green color until the WT phenotype was restored. At the heading stage, the *wlp3* panicle exhibited the albino phenotype again (Fig. 1C and D). Compared to WT, the panicle was longer, the thousand-grain weight was lower, and the number of grains per panicle was higher, which may be related to the increased ear length and tiller number (Fig. 1E–I). In addition, the leaf color of *wlp3* was affected by temperature, but the leaves recovered to a WT-like phenotype at 24 °C and the chlorophyll content in *wlp3* was almost the same as WT (Fig. 2A, D, and E). However, *wlp3* exhibited an obvious albino phenotype at 28 and 33 °C and the chlorophyll content was significantly lower compared to WT suggesting that this is a temperature-sensitive mutant (Fig. 2B–C, G–J).

### *wlp3* Affects Chloroplast Development

**Fig. 3:**
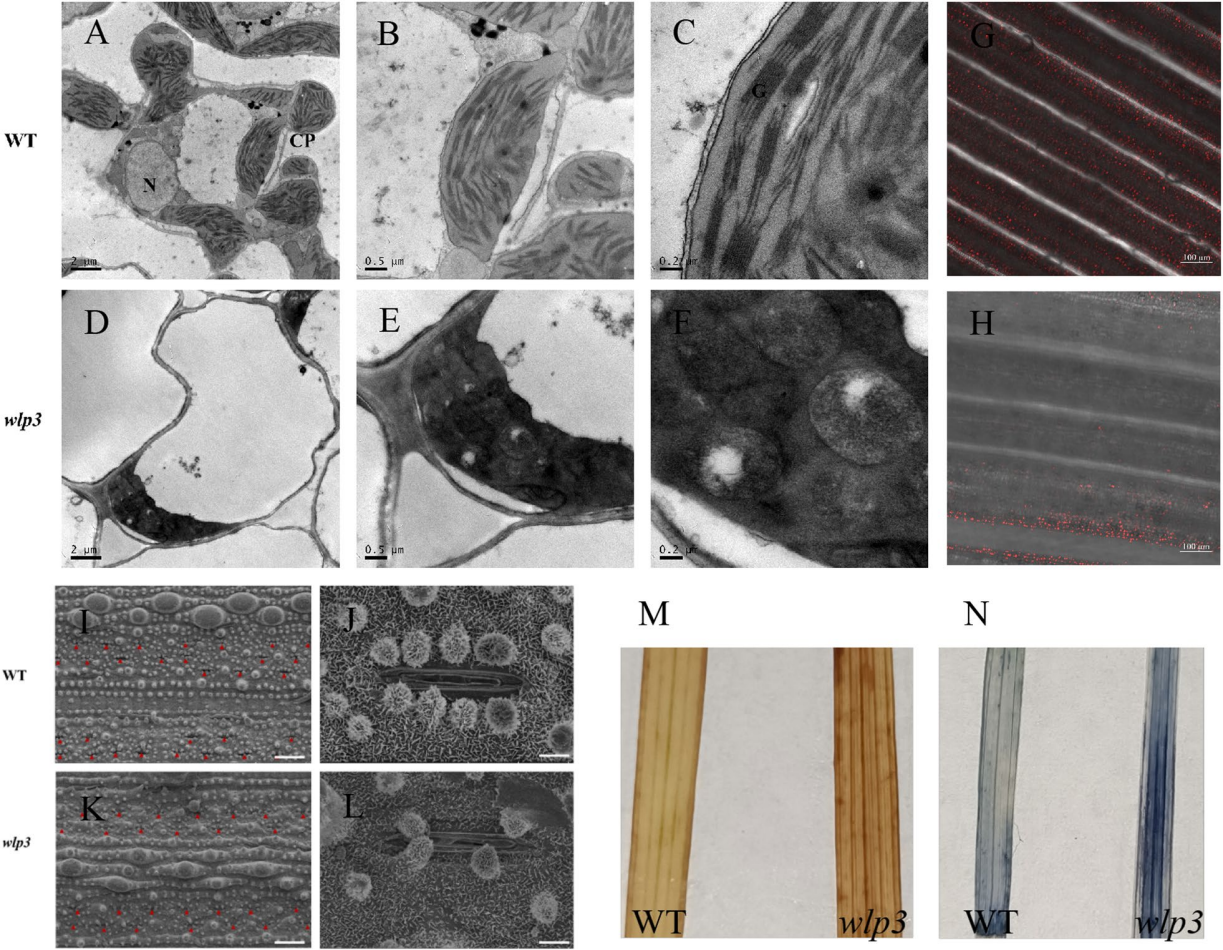
TEM, SEM, and laser confocal microscopy of WT and *wlp3* leaves. **A**–**C** Ultrastructure of the WT chloroplast. **D**–**F** Ultrastructure of *wlp3* chloroplast, where N is the nucleus and CP is the chloroplast. **G** Chloroplast autofluorescence in WT leaves. **H** Chloroplast autofluorescence in *wlp3* leaves. **I**–**J** SEM of WT (scale bar = 150 nm in I and 15 nm in J). **K**–**L** SEM of *wlp3* (scale bar = 150 nm in K and 15 nm in L). **M**–**N** DAB and NBT staining of WT and *wlp3*

To examine the effect of the *wlp3* mutation on chloroplast structure, the ultrastructure of the green and white parts of WT and *wlp3* plants was observed by transmission electron microscopy (TEM). In WT, the chloroplast developed normally and the thylakoid structure was ordered (Fig. 3A–C). In the albino part of *wlp3*, the chloroplast did not reach maturity since the structure of the middle chloroplast was abnormal (Fig. 3D–F). Furthermore, the WT and *wlp3* leaves were also observed by laser confocal microscopy. The chloroplast autofluorescence was reduced in *wlp3*, suggesting that the *wlp3* mutation can cause abnormal chloroplast development.

The leaf surface structures of WT and *wlp3* at seedling stage were observed by scanning electron microscope (SEM). The silicoid papillae around the stomata, the number of stomata, and the stomatal openings were all reduced and the pores were narrowed in *wlp3* compared with WT (Fig. 3J–L).Diaminobenzidine (DAB) and Nitrotetrazolium Blue chloride (NBT) staining demonstrated a large number of precipitated blue and brown particles, respectively, indicating a large accumulation of superoxide anion and superoxidase in *wlp3* leaves. Since the accumulation was mainly near the leaf veins, which coincides with the albino parts on *wlp3* leaves, the damage to the chloroplast structure in *wlp3* may cause the accumulation of peroxidase and superoxide anion (Fig. 3M and N).

To investigate whether if the *WLP3* mutation can cause changes in the rate of chlorophyll synthesis, WT and *wlp3* seeds were grown in the dark for seven days and then transferred to a light incubator. The chlorophyll content of leaves was measured. The chlorophyll synthesis rate was faster at 6 h of light, with no significant difference between WT and *wlp3.* WT chlorophyll synthesis rate was higher compared to WT from 24 h. From 36 to 48 h, the chlorophyll synthesis rate of *wlp3* increased significantly, reducing the gap between the WT. However, the rate in *wlp3* was always lower than in WT. In conclusion, the *WLP3* mutation caused the reduction of chlorophyll synthesis and its accumulation in *wlp3* (Additional file 2: Fig. S1).

### Map-Based Cloning of *WLP3*

The albino white spike phenotype was not detected in any F_1_ plants. Self-crossing of the F_1_ generation produced F_2_ progeny with a white-leaf and white-panicle phenotype similar to *wlp3*. Further analysis indicated that the ratio of the green to the white-leaf and white-panicle phenotypes was close to 3:1. The chi-squared test indicated that the *wlp3* phenotype was controlled by a recessive gene (Additional file 1 Table 1).

**Fig. 4:**
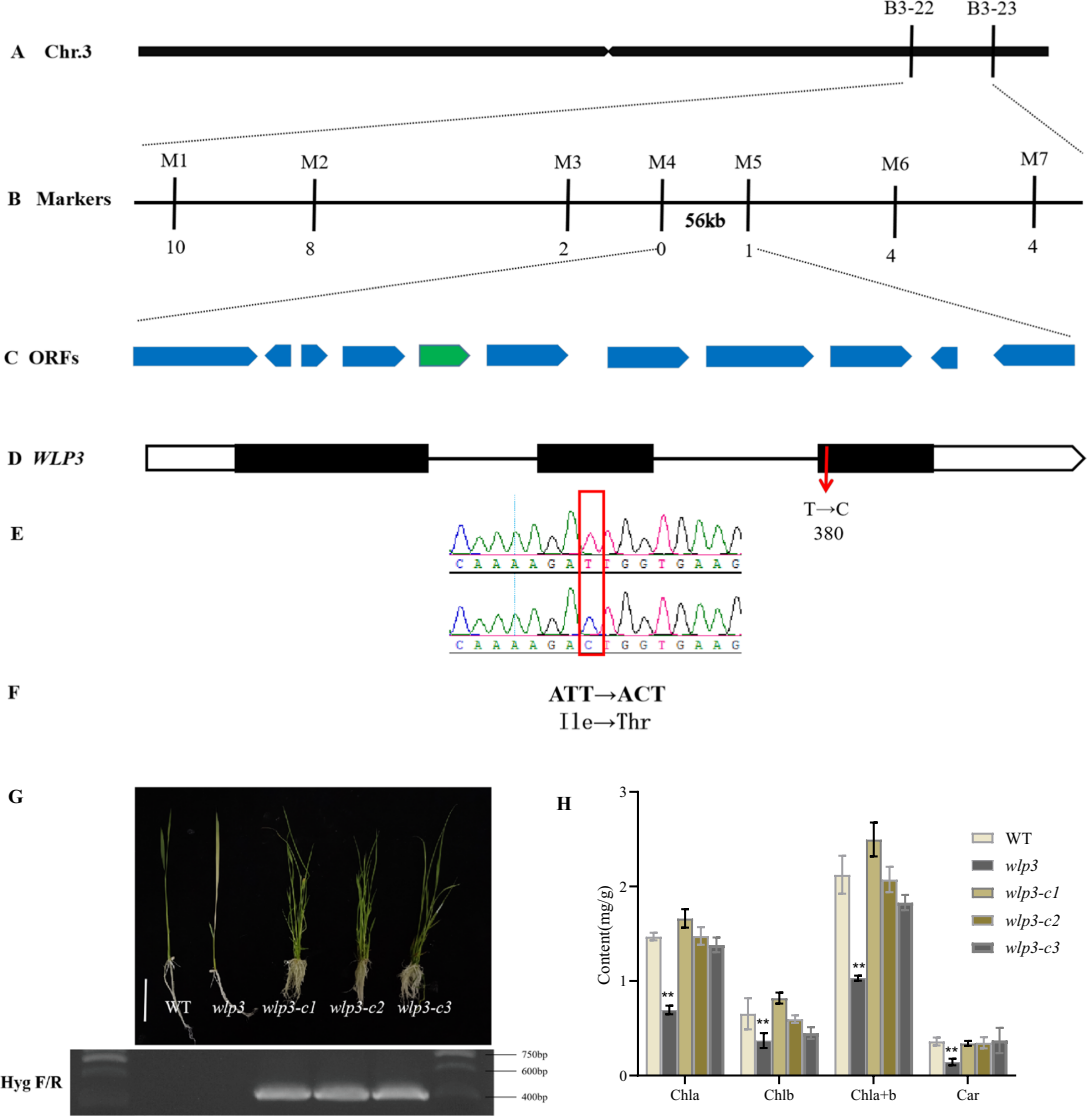
Map-based cloning of the *WLP3* gene. **A** Preliminary mapping of the *wlp3* mutant using 21 recessive F_2_ plants with 268 SSR markers. The gene mapped to a region between molecular markers B3-22 to B3-23 on chromosome 3*.* **B**-**C** Fine mapping of *WLP3* further localized the mutation to the 56 kb genomic region between M4 and M5. **D** *WLP3* gene structure. Red arrows indicate mutation site. **E**–**F** Mutation sites. **G** Functional complementation of *wlp3.* Transgenic plants were verified by the presence of the hygromycin selectable marker gene. **H** Chlorophyll content of complementary lines

A single F_2_ plant with the albino phenotype was used to map the *WLP3* gene. The results of the initial mapping showed that *WLP3* was located between the molecular markers B3-22 and B3-23 on Chromosome 3 (Fig. 4A). A new indel marker was then developed and *WLP3* was finally located within a physical distance of 56 Kb between the M4 to M5 indel markers (Fig. 4B**)**. Using the Rice Genome Browser (http://rice.uga.edu/cgi-bin/gbrowse/rice/), 11 open reading frames (ORFs) were identified to be located within this interval (Fig. 4C). Sequencing of these ORF-specific primers revealed that the third exon of the *LOC_Os03g61260* gene contained a mutation at position 380 of the coding region where the T base was mutated to C (Fig. 4E). This transition mutation resulted in the final translation of the amino acid from isoleucine to threonine (Fig. 4F). Therefore, *LOC_Os03g61260* was selected as the candidate gene for *WLP3*.

To verify whether the *WLP3* mutation results in the white leaf and white panicle, *Agrobacterium*-mediated transformation was used to transform pCAMBIA1300-*WLP3*, containing a 700 bp promoter upstream of *WLP3*, into the *wlp3* calli. Through phenotypic screening, 19 transgenic plants were successfully transformed and all exhibited a normal phenotype (Fig. 4G). The chlorophyll content of the complementary lines was then examined showing that all returned to the WT level (Fig. 4H**)**. Genetic complementation of *WLP3* confirmed that *LOC_Os03g61260* was the *WLP3* gene. The single-base mutation in this gene resulted in the albino phenotype in rice at the seedling stage,which returned to the green color at later developmental stages.

### *WLP3* is Expressed in Leaves and Panicles

**Fig. 5:**
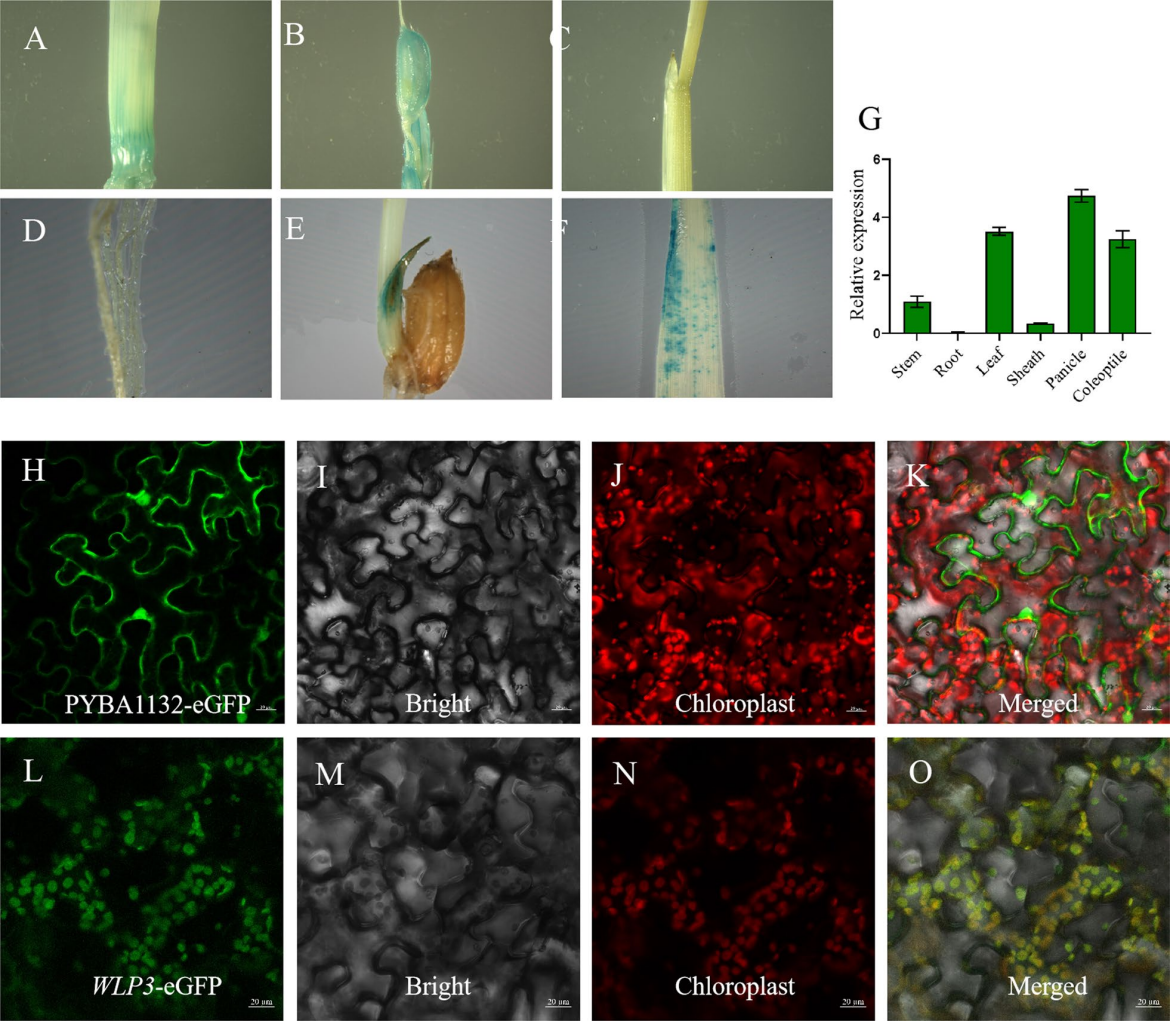
Time–space expression of *WLP3.* **A** stem, **B** panicle, **C** sheath, **D** root, **E** coleoptile, **F** leaf. **G** *WLP3* expression in different tissues. **H**- **K** PYBA1132-eGFP transiently expressed in tobacco. **L**-**O** *WLP3* transiently expressed in tobacco

*WLP3* expression in different parts of the plant was analyzed by GUS staining. *WLP3* was almost not expressed in mature leaves and leaf sheaths (Fig. 5C**)**, but was most highly expressed in panicles (Fig. 5B**)**, followed by stems (Fig. 5A**)**, and also in coleoptiles (Fig. 5E**)**. Staining of GUS transgenic plants at the seedling stage showed that *WLP3* expression was higher in leaves at the seedling stage. Real-time quantitative PCR (qRT-PCR) was used to detect *WLP3* expression at different stages and in different tissues. The detection results were consistent with GUS staining, further clarifying the cause of *wlp3* leaves changing from white at the seedling stage to green at the later developmental stages (Fig. 5G**)**. At the tillering stage, the transcript level decreases because *WLP3* no longer plays a role. *WLP3* expression increases in the panicle at the heading stage. Therefore, mutation of *WLP3* gene causes the albino phenotype of *wlp3* in leaves at the three-leaf stage and panicles at the heading stage..

### Subcellular Localization of *WLP3*

Subcellular localization of *WLP3* was investigated using ZH11 cDNA as the template, the target fragment was amplified using WLP3-Ecoli1-F and WLP-Kpn1-R, and ligated to PYBA1132-eGFP by infusion. The constructed 1132-WLP3-eGFP and PYBA1132-eGFP empty vectors were transferred into EHA105 cells for transient transformation in tobacco. The fluorescence position of the empty vectors was observed at different locations in the cell using laser confocal microscopy (Fig. 5H–K). *WLP3* fluorescence coincided with chloroplast autofluorescence, indicating its expression in chloroplasts (Fig. 5L–O).

### Phylogenetic Analysis of *WLP3*

**Fig. 6:**
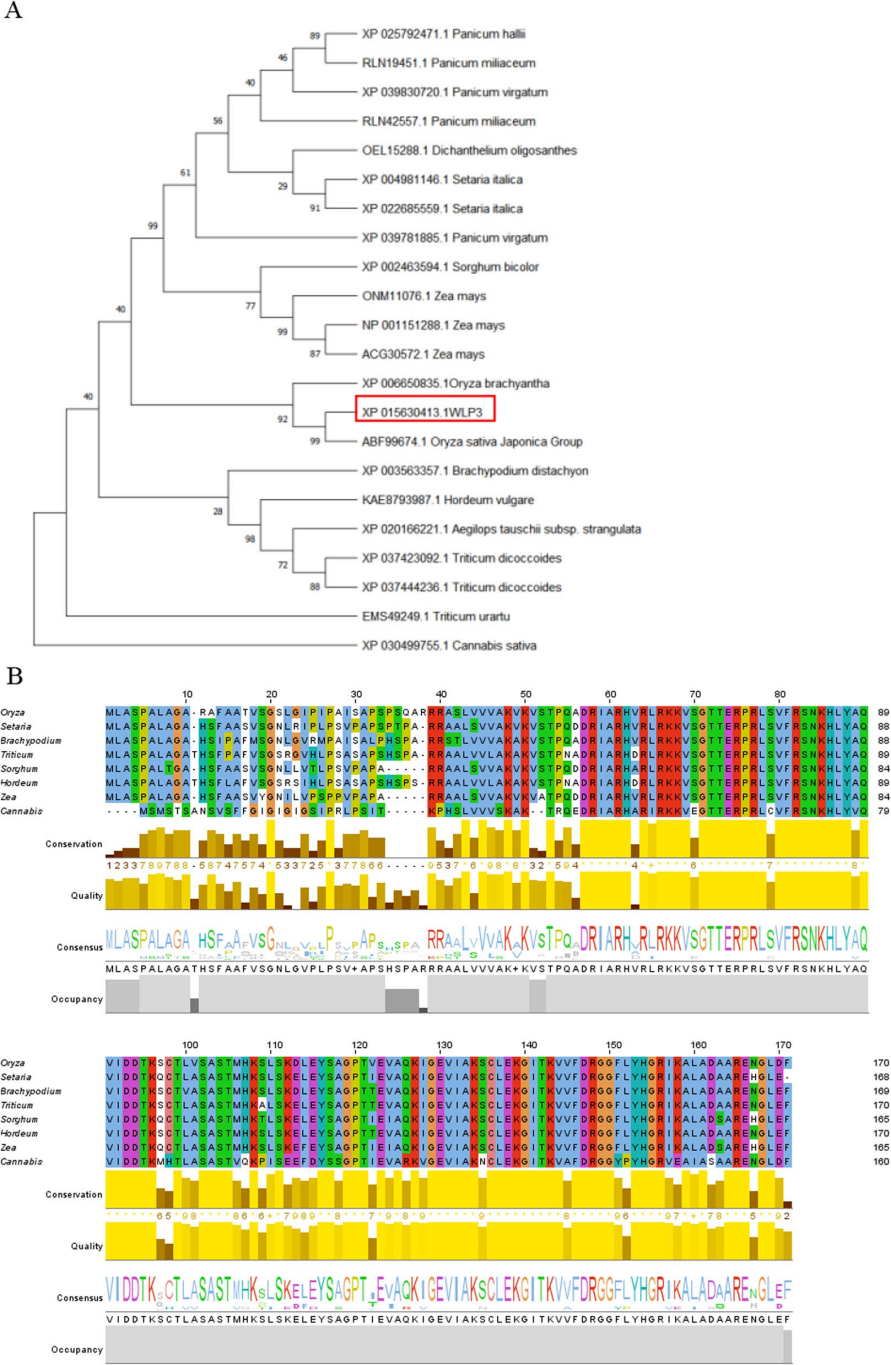
Bioinformatics analysis of *WLP3*. Bioinformatics analysis of *WLP3.* **A** Phylogenetic tree of *WLP3*

Homologous sequences were identified using phylogenetics in the genomes of rice, maize, *Brachypodium*, barley, and other plants (Fig. 6A). The signal peptide site (https://services.healthtech.dtu.dk/services/SignalP-5.0/) predicted a chloroplast signal peptide at amino acids 1–21 (Additional file 2: Fig. S2A) and the SMART site (http://smart.embl-heidelberg.de/) predicted the presence of a ribosomal L18p domain from amino acids 51 to 170. L18 (L5e) was a ribosomal protein located in the central protrusion of the large subunit (Additional file 2: Fig. S2B).

The SWISS-MODEL website was used to predict the structure of the WLP3 protein. 3 α-helices and 1 β-sheet were identified on the *WLP3* protein and the *wlp3* mutation site was located in the α on the spiral (Additional file 2: Fig. S2C). The results of multiple sequence alignment showed large differences in the first 50 amino acids in the WLP3 sequence, while the latter amino acids had small differences among different species, and the amino acids constituting the domain were more conservative (Fig. 6B). These results suggest that WLP3 domains have important organismal functions and are therefore highly conserved during evolution.

The genetic information in the biological genome is first transferred to mRNA by RNA polymerase. The mRNA is then translated by ribosomes to encode the biologically active proteins and perform corresponding functions. GO enrichment and Rice FREND analyses revealed that WLP3 was mainly co-expressed with protein translation, protein biosynthesis, organic acid metabolism and isogenic groups (Additional file 2: Fig. S2D), suggesting that WLP3 is largely responsible for macromolecule (i.e., proteins) synthesis and plays a central role in normal rice growth.

### *WLP3* Mutation Affects the Expression of GENES Related to Chloroplast Development

**Fig. 7:**
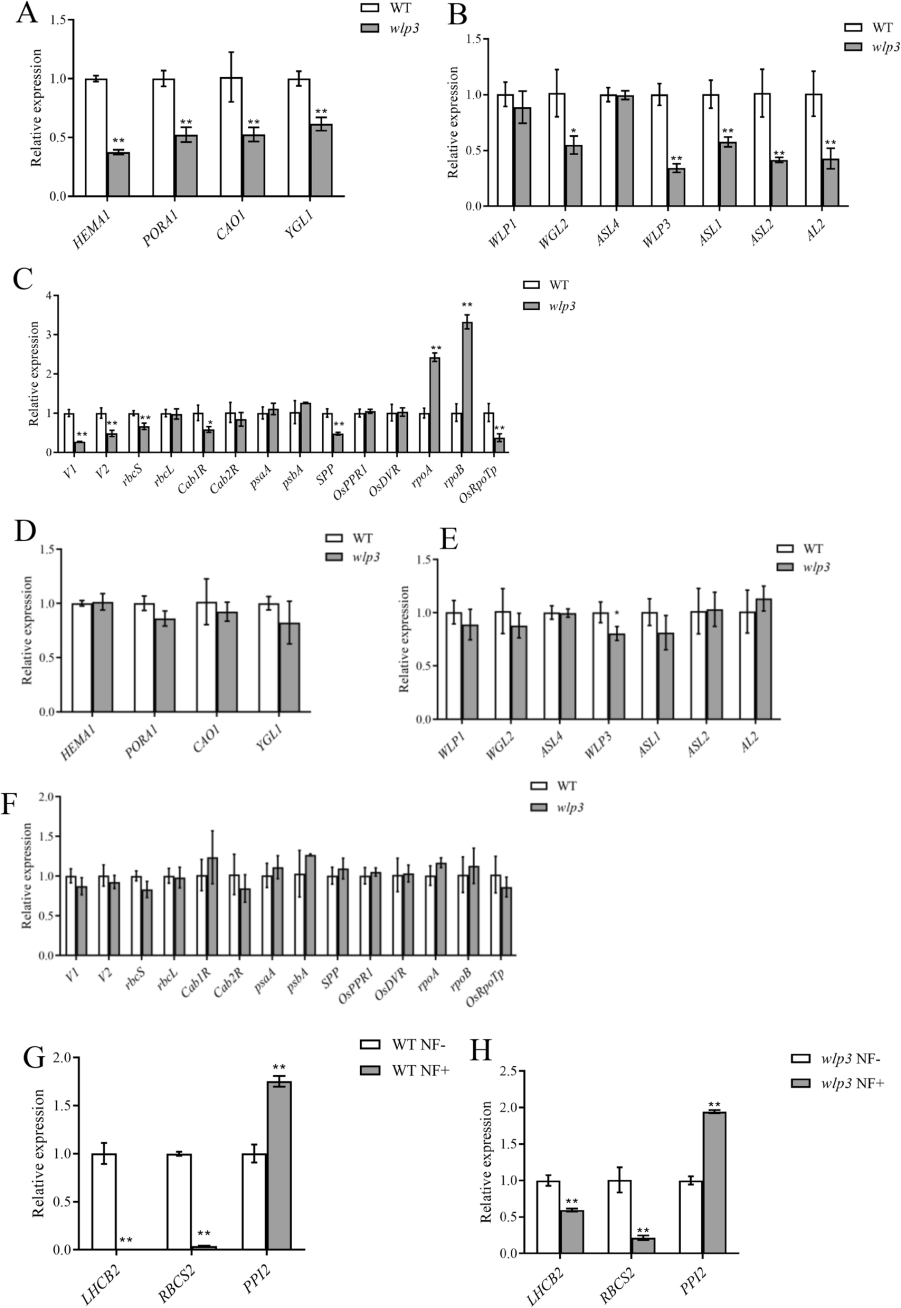
**A** Genes related to chlorophyll synthesis at 28 ℃. **B** Ribosomal gene-related genes at 28 ℃. **C** Genes related to chloroplast development at 28 ℃. **D** Genes related to chlorophyll synthesis at 23 ℃. **E** Ribosomal gene-related genes at 23 ℃. **F** Genes related to chloroplast development at 23 ℃. **G** Related genes in the WT before and after fluoxamin treatment. **H** Related genes in *wlp3* before and after fluoxapyr treatment

qRT-PCR was used to examine the relevant gene expression at different temperatures in WT and mutant three-leaf stages trifoliate leaves. The results showed that genes related to chlorophyll synthesis in *wlp3*, including *HEMA1*, *PORA1*, *CAO1,* and *YGL1* (Fig. 7A) and some genes related to ribosomal protein synthesis, such as *WGL2*, *ASL2,* and *AL2* (Fig. 7B) were significantly down-regulated at 28 ºC. Detection of genes related to chloroplast development revealed that the expression of RNA binding protein gene *V1*, guanyl kinase gene *V2*, RUBP carboxylase small subunit *rbc*, PSII light-harvested chlorophyll A/B binding protein *Cab1R*, and *SPP* expression was significantly downregulated. *OsRpoTp*, the gene encoding NEP, was significantly downregulated, while *rpoA* and *rpoB*, the genes encoding PEP, were significantly upregulated. In rice, NEP contains only one central subunit encoded by *OsRpoTp*, whereas PEP contains four small subunits encoded by *rpoA* and *rpoB* genes. The *WLP3* mutation resulted in a decreased transcript level of NEP and an increased transcript level of PEP-related genes (Fig. 7C). However, at 23 ºC, the expression of genes related to chloroplast development and chlorophyll synthesis in *wlp3* was restored to WT levels (Fig. 7D-F). These results suggest that the recovery of leaf color in *wlp3* was caused by the recovery of mRNA expression in the plant at low temperatures.

Recent studies have shown that when chloroplast growth and development is inhibited, special signaling substances are released from the chloroplasts to the nucleus, reducing the expressiom of chloroplast protein encoded by the nucleus. In this way, cells are adapted to chloroplast development. This is achieved by the nucleocytoplasmic retrograde signaling (RS) pathway. However, when certain genes are mutated, the nucleocytoplasmic retrograde signal is blocked, and the translation level of nuclear-encoded chloroplast proteins is not affected by the chloroplast developmental signal. Norflurazon (NF) was therefore used to treat the three-leaf stage to determine whether the *WLP3* mutation could lead to blockage of nucleocytoplasmic retrograde signaling and whether *wlp3* may cause genome uncoupling at the molecular level. Fluroxypyr may inhibit nuclear gene expression via the tetrapyrrole biosynthetic pathway. *LHCB2* and *RBCS2*, two key target genes of nucleocytoplasmic retrograde signaling, were analyzed by qRT-PCR useing the retrograde signaling gene *PPI2* as a control. The results showed that *LHCB2* and *RBCS2* were not expressed in WT after treatment with fluroxypyr, while their expression significantly decreased *wlp*3 after treatment. The decrease in *wlp3* was much less compared to WT. The level of change of *PPI2* in *wlp3* was consistent (Fig. 7G–H) suggesting that nucleocytoplasmic retrograde signaling was blocked in *wlp3*,which displays a partial genome uncoupling phenotype.

### *wlp3* is Insensitive to ABA and Abiotic Stress Response

**Fig. 8:**
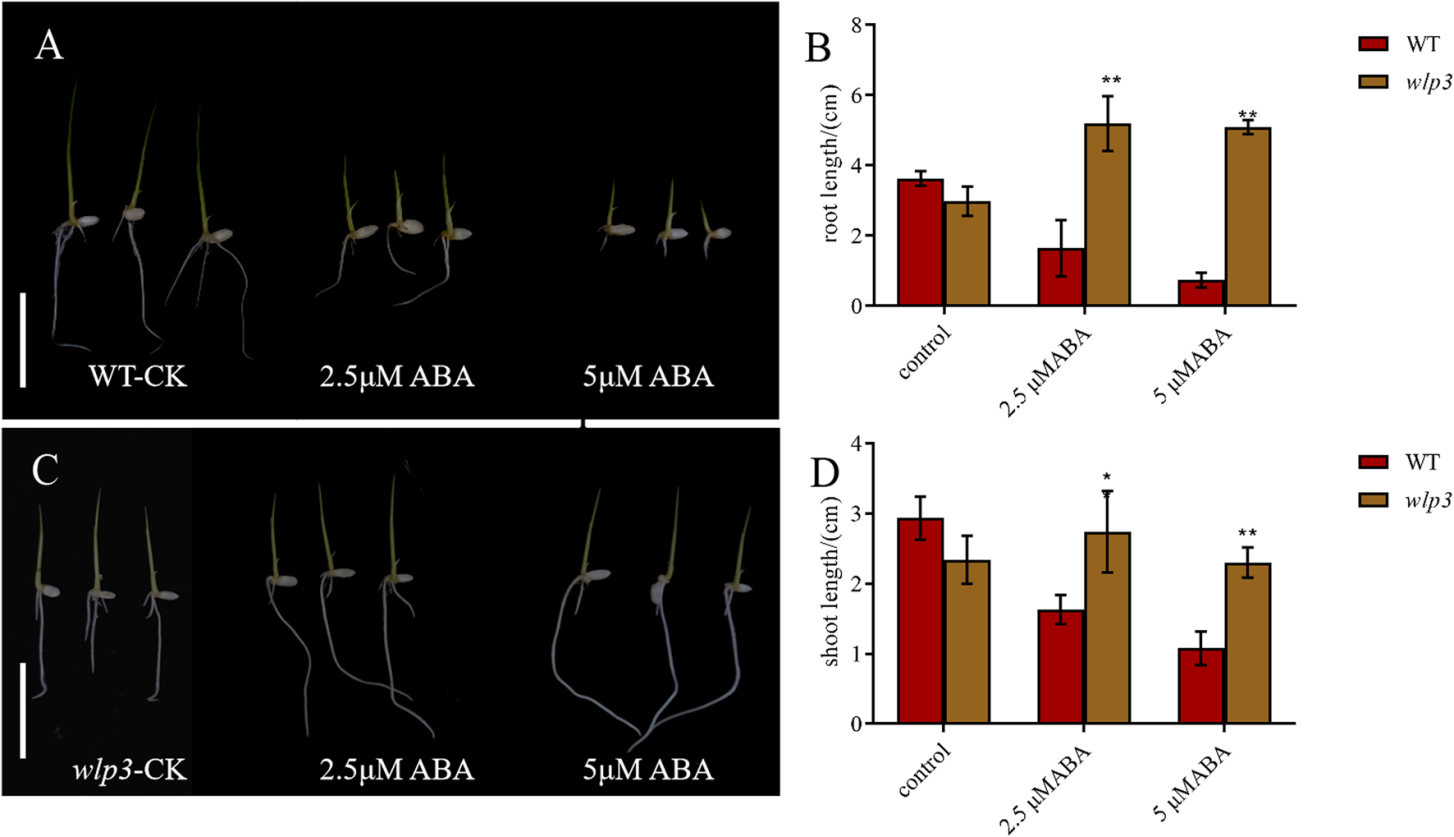
Response of WT and *wlp3* to ABA and cold stress. **A** Phenotypic diagram of WT under different ABA concentrations (scale bar = 3 cm). **B** The stem and root length of WT treated under different ABA concentrations (scale bar = 3 cm). **C** Phenotypic plots of *wlp3* under different ABA concentrations. **D** Stem and root lengths of *wlp3* after different ABA treatments

To determine whether the *WLP3* mutation affects the sensitivity of plants to ABA, WT and *wlp3* were inoculated on 1/2MS medium. Root and stem lengths were measured after seven days. Both the stems and roots of WT were significantly inhibited on the 2.5 μM medium, with a more pronounced inhibition on the 5 μM medium (Fig. 8A-B). However the root and stem lengths of *wlp3* significantly increased on the 2.5 μM medium compared with the control group but slightly decreased on the 5 μM medium compared with 2.5 μM (Fig. 8C-D). These results suggest that ABA treatment at 2.5 and 5 μM promoted the growth of *wlp3*, but more so at 2.5 μM. Furthermore, the *WLP3* mutation reduced the sensitivity of the plant to ABA.

To determine whether *wlp3* is involved in drought and salt stress responses in rice, WT and *wlp3* seeds were grown in a nutrient solution containing 20% PEG6000 and 1/2MS medium containing 100 mM NaCl. Salt tolerance-related gene expression was analyzed by measuring the root and stem lengths of WT and *wlp3* after ten days of growth. RNA was extracted from WT and *wlp3* plant at this time. The results showed that the root length of *wlp3* under salt treatment was significantly lower than that of WT (Additional file 2: Fig. S3A–D), indicating that the sensitivity of *wlp3* under 100 mM NaCl stress was higher. The expression levels of drought and salt tolerance-related genes were significantly higher than those of *wlp3* (Additional file 2: Fig. S4A–F) suggesting that *wlp3* was more sensitive to drought and salt stress.

### WLP3 Interacts with other Ribosomal Proteins

**Fig. 9:**
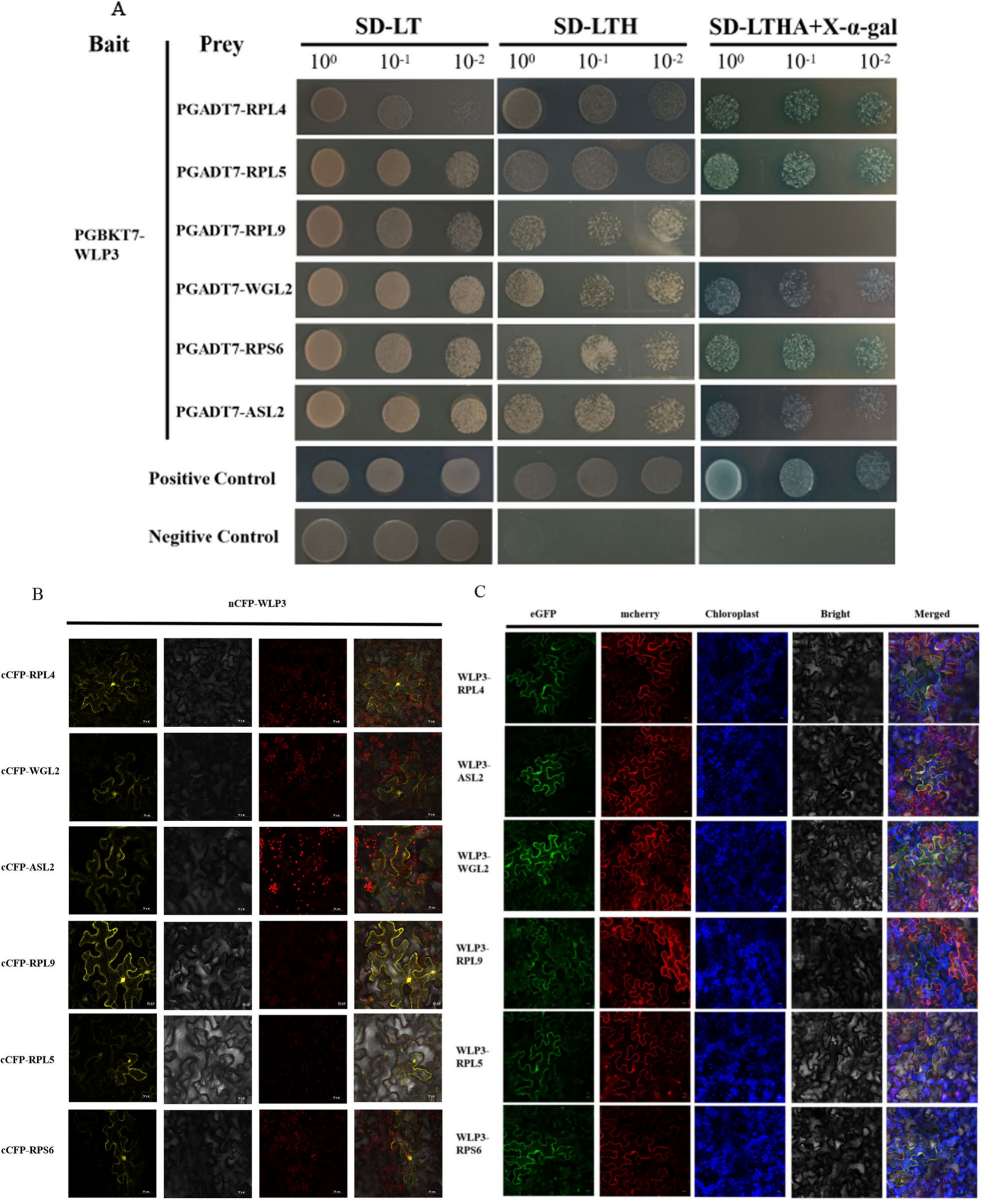

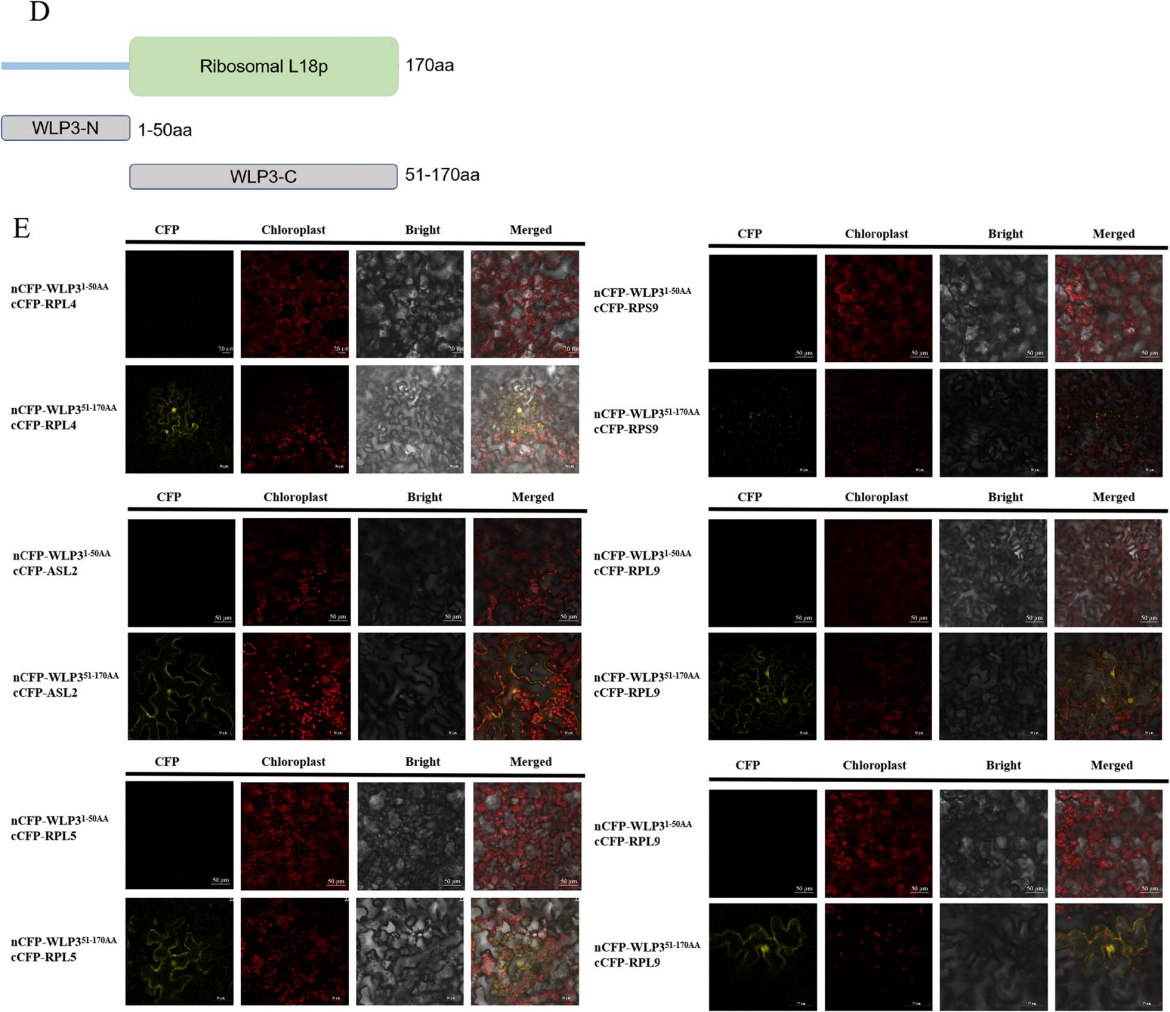
WLP3 interacts with other ribosomal subunits. **A** Y2H analysis of WLP3. In yeast cells, WLP3 is associated with RPL4,RPL5,RPL9,WGL2,RPS6 and ASL2, respectively. **B** Verification of BiFC of WLP3.In tobacco cells, WLP3 interacts with other ribosomal subunits in the cytoplasm and nucleus. **C** Co-localization of WLP3 with other ribosomal subunits.WLP3 interact with other ribosomal subunit WLP3-EGFP fluorescence from the chloroplast transfer to the cytoplasm and nucleus. **D** Schematic representation of the truncated protein structure of WLP3. **E** Interaction analysis of the truncated WLP3 with other ribosomal proteins

The ribosome is a dense ribosomal protein particle that can dissociate into two subunits, each containing different grouped ribosomal subunits. To verify whether WLP3 interacts with other ribosomal subunits, the *WLP3* coding region was constructed into PGBKT7 and the coding regions of RPL4, WGL2, ASL2, RPL9, RPL5, RPS6 was constructed into PGADT7, using the yeast two-hybrid (Y2H) method. WLP3 and PGADT7 could not grow on the triple and quadruple dropout supplements, indicating that WLP3 does not have self-activating activity. However, WLP3 and RPL4, RPL5, RPS6, ASL2, and WGL2 could grow on the quadruple dropout supplements and RPL9 on the triple dropout supplements (Fig. 9A). These results suggest that WLP3 and RPL4, RPL5, RPS6, ASL2, and WGL2 exhibit strong interactions, while RPL9 has a weaker interaction.

A bimolecular fluorescence complementation (BiFC) assay was used to verify whether WLP3 interacts with other ribosomal proteins in planta assay. The recombinant plasmids containing WLP3-nCFP and other cCFP plasmids were co-injected into tobacco leaves. Using fluorescence confocal microscopy, the tobacco leaves co-injected with WLP3, RPL4, WGL2, ASL2, RPL9, RPL5, and RPS6 all showed fluorescence and the interactions occurred in both the nucleus and cytoplasm (Fig. 9B). Construction of the *wlp3* single-base substitution on nCFP showed that the interaction of *wlp3* with other ribosomal subunits was not affected (Additional file 2: Fig. S5 A).

In BiFC experiments, the fluorescence produced by the interaction of WLP3 with ribosomal subunits was detected in chloroplasts,cytoplasm and nucleus. Therefore, it is hypothesized that the interaction of WLP3 with ribosomal subunits. Initially, RPL4, WGL2, ASL2, RPL9, RPL5, and RPS6 cDNA were constructed into the PYBA-1132-eGFP vector. All subunits were localized within the chloroplasts by *Agrobacterium*-transformed tobacco (Additional file 2: Fig. S5B). Subsequently, RPL4, ASL2, RPL9, RPL5, and RPS6 cDNA were constructed into the PYBA1138-mcherry vector and transferred into *A. tumefaciens* and then co-injected into tobacco with PYBA1132-WLP3-eGFP. GFP fluorescence was translocated from the chloroplast to the cytoplasm and nucleus (Fig. 9C). Different ribosomal subunits constructed at the cCFP in BiFC with PYBA1132-WLP3-eGFP were co-injected to eliminate the effect of the mcherry protein. Fluorescence was also distributed throughout the cell (Additional file 2: Fig. S5C). These results suggest that the interaction of WLP3 with other ribosomal subunits does indeed affect the localization of WLP3.

To identify the key domains with which WLP3 interacts in these proteins, WLP3 was divided into two distinct parts (Fig. 9D): the N-terminal (1-50aa) and the C-terminal (51-170aa). The truncated fragments containing only part WLP3-N or WLP3-C of the C-terminal domain were cloned and attached to nCFP vectors which were then co-transformed with cCFP-RPL4, WGL2, ASL2, RPL9, and RPL5 in EHA105. The results demonstrate that RPL4, WGL2, ASL2, RPL9, RPL5 and RPS6 can only interact with WLP3-C. However, WLP3 could not interact with other truncated domains (Fig. 9E).

## Discussion

Rice leaf color mutants are useful for studying chloroplast development because their phenotypic differences are esay to detect. There are many types of leaf mutations, with albino being the most severe phenotype,in whichthe leaves are completely damaged. These leaves contain on photosynthetic pigments and cannot perform photosynthesis normally. Therefore, the albino mutant is lethal to the growth and development of rice.

The albino white spike mutant in this study was induced by EMS mutagenesis from the rice variety ZH11. Only the *wlp3* mutant exhibited the white stripe phenotype before the three-leaf stage. The white stripes were distributed along the leaf veins on both sides of the large and small veins. The newly emerging leaves at the four-leaf stage reverted to the normal phenotype. The albino phenotype was affected by temperature, with alleviation at 24 °C. A single base mutation was found in the *LOC_Os03g61260* gene by map-based cloning. Using a complementation experiment, the leaf color of *wlp3* recovered the same phenotype as WT, demonstrating that *LOC_Os03g61260* was a *wlp3* mutant. These results also suggest that *WLP3* is a gene that affects rice leaf color and plays an important role in rice growth and development. Results of GUS staining showed that *WLP3* expression was mostly higher in the chloroplasts, where WLP3 is localized, of three-leaf stage leaves and the panicle just heading. *ASL1* was the first chloroplast ribosomal protein mutant gene identified, encoding the small S20 subunit of the ribosomal protein, which also showed an albino phenotype. Unlike *wlp3*, the *asl1* phenotype is seen throughout the plant whether at low or high temperatures. Since *asl1* cannot perform normal photosynthesis, the plants die within a month of germination due to their inability to synthesize nutrients independently. Similar to *ASL1, AL1* (encoding the large ribosomal protein subunit L12), *ASL2* (encoding the large ribosomal protein subunit L21), and *ASL4* (encoding the small ribosomal protein subunit S1) the plants are albino, cannot grow normally, and are lethal at the seedling stage. It is speculated that these genes have unique and irreplaceable roles during rice growth and development. However, *WLP1* (encoding ribosomal protein large subunit L13), similar to *WLP3* in this study, is a temperature-sensitive mutant. The albino phenotype gradually disappears as the rice growth to the heading stage. This has a white spike phenotype, but unlike *wlp3*, *wlp1* is a low-temperature sensitive mutant, with the albino phenotype aggravated at low temperatures. However, high temperature can alleviate the albino phenotype. Therefore, it is speculated that *WLP3* is a weak mutant like *WLP1.* During the life cycle of rice, only the development of chloroplasst before the three-leaf stage plays an important role, while other proteins are required only in the subsequent biological process, and therefore the green phenotype is restored in later stages of growth and development.

In *E. coli*, RPL18 plays an essential role in the biological assembly of ribosomes in vivo*.* Mutations in this gene result in reduced mRNA translation capacity. In addition, RPL18 plays a critical role in bacteria, protists, yeast, humans, and other plants. However, the role of RPL18 in plants remains poorly understood. *WLP3* was identified as a chloroplast ribosomal protein large subunit L18, with a signal peptide in the first 21 amino acids of the protein and a ribosomal L18p structure from the 51st to the 170th amino acids. Phylogenetic and multiple sequence alignment analyses revealed that *WLP3* domains are conserved in higher plants, suggesting that this domain may play an important role in many plants.

The development of plastid into photosynthetically active chloroplasts is controlled by both NEP and PEP (Hedtke et al. 1997). Genes related to chlorophyll synthesis, chloroplast development, and ribosomal protein synthesis were detected by qRT-PCR. The results showed that the transcript level of the NEP gene *RpoTp* decreased, while the expression levels of genes responsible for chloroplast genome transcription (i.e., *rpoA* and *rpoB*) transcribed by NEP were significantly increased. No significant difference was detected in the expression of PEP-transcribed proteins (i.e., *rbcL*, *psaA*, *psaB,* and others), suggesting the existence of other regulatory pathways in plants to replace some NEP genes when they cannot be expressed normally. Nevertheless, nuclear-encoded chlorophyll synthesis genes were significantly reduced in *wlp3*.

Furthermore, the expression of some ribosomal protein subunits were decreased in *wlp3*, suggesting that *WLP3* mutations may affect the normal assembly of ribosomes in chloroplasts, resulting in the failure of translation of some photosynthesis-related genes into proteins after transcription. The *WLP3* mutation blocked the nucleocytoplasmic retrograde signaling, which affected the transcription of chloroplast-related genes encoded in the nucleus and ultimately causing the color change of *wlp3* leaves. *Arabidopsis WLP3* homolog *AtPRPL18* is required for plant development. Knockdown of *AtPRPL18* delays the transition from the *Arabidopsis* spheroid stage to the cardiac stage (Chen et al. 2022).

Previous studies have shown that ribosomal proteins can play an important role in plants under biotic and abiotic stresses. RPS13, RPS6, and RPL37 in soybean were shown to be induced by a low-temperature environment, demonstrating the low-temperature stress response of these three ribosomal proteins (Kim et al. 2014). Overexpression of RPL13a in eggplant increase potato resistance to Verticillium wilt (Yang et al. 2013). In tobacco, mutations in RPL12 and RPL19 result in reduced plant resistance to bacterial pathogens (Nagaraj et al. 2016). Overexpression of RPL23a in transgenic rice can improve rice water use efficiency, thereby increasing rice tolerance to drought and salt stress (Cherepneva et al., 2013). Furthermore, the response of *wlp3* to drought and salt stress was investigated. The results showed that *wlp3* leaf tips wilted under drought stress. Compared to WT, the expression of *wlp3* and drought tolerance-related genes decreased, suggesting that this mutation increases drought sensitivity and thereby decreases tolerance. Under salt stress, the expression of salt-tolerance-related gene was slightly increased in the WT compared to *wlp3*, suggesting that the WT was more salt-tolerant. Rice is very sensitive to salt concentration during its growth, and high salt ion concentration can reduce rice yield. Therefore, improving the salt tolerance of rice may help to increase rice yield. To study the molecular mechanism of wlp3 in regulating salt tolerance, it is theoretically helpful to improve the breeding level of rice varieties with salt tolerance.

**Fig. 10:**
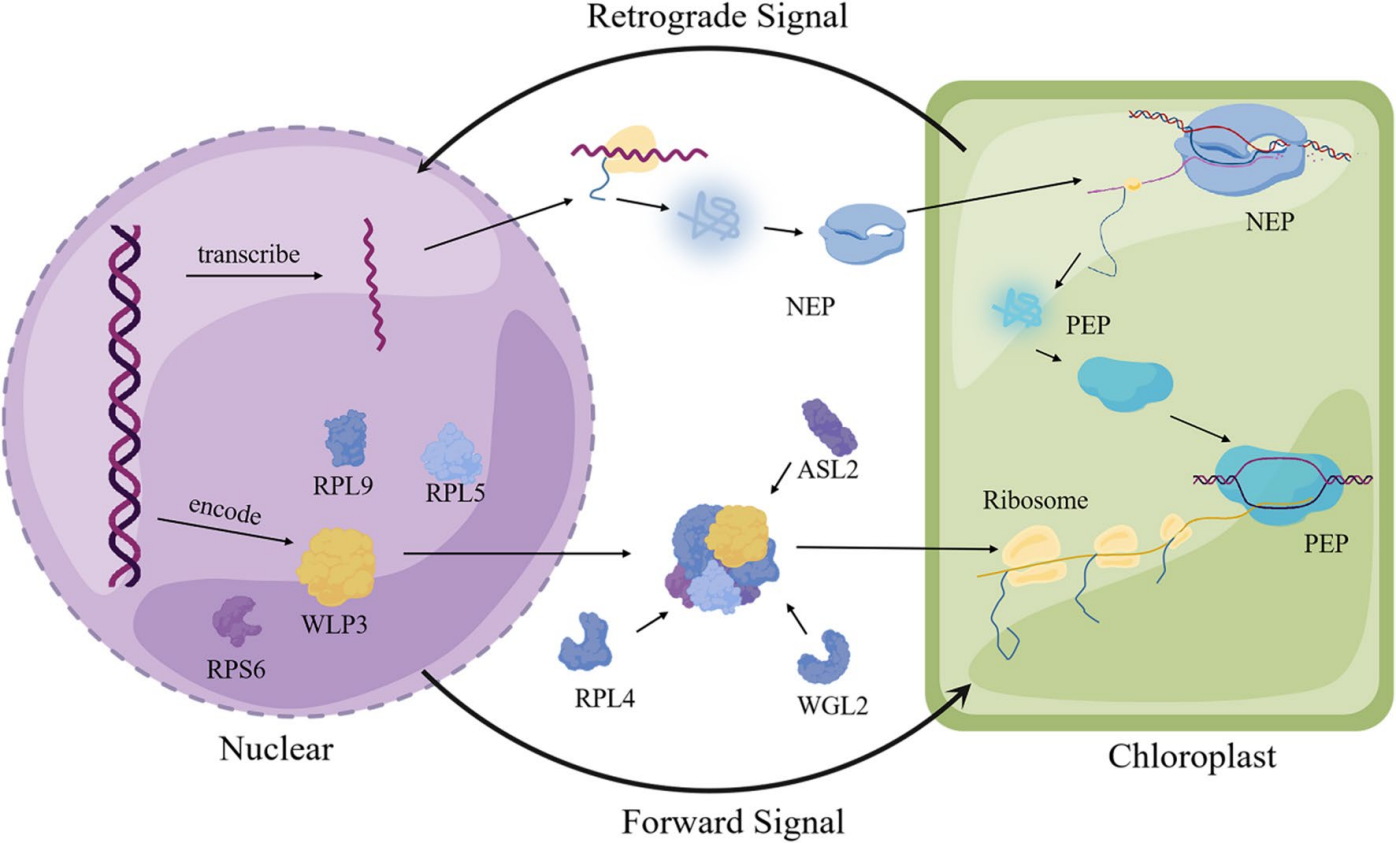
Putative *WLP3* pathways affecting rice leaf color

In the cytoplasm and organelles of plant cells, ribosomes are crucial for the precise regulation of plant growth and development. In bacteria, *wlp3* homolog is L18, and it induces a conformational change by binding to 5S rRNA, which induces L5 to eventually bind to 5 s rRNA (Kim et al. 1995). Therefore, the successful binding of L18 and L5 to 5 s rRNA plays a critical role in the normal assembly of the ribosome. This study showed that WLP3 can interact with RPL4, WGL2, ASL2, RPL9, RPL5, and RPS6 to assemble into chloroplast ribosomes with normal functions. BiFC was also used to verify the yeast double complex results. Furthermore, the interaction position of WLP3 with other ribosomal subunits in BiFC was different from the subcellular localization. Mammalian RPS3 with endonuclease activity is involved in DNA damage repair (Kim et al. 1995). Therefore, it was hypothesized that WLP3 to bind to other ribosomal proteins and perform other biological functions in the cytoplasm or nucleus. According to the above experimental results, a mechanism of action of WLP3 was deduced: WLP3 is first transcribed and translated by nuclear genes, assembles with other ribosomal subunits in cells, and enters chloroplasts to translate the genes related to leaf color and photosynthesis transcribed by the chloroplast genome (Palm et al. 2018). When WLP3 is mutated, the genes in the chloroplast cannot be translated normally, ultimately causing the change in rice leaf color (Fig. 10).

## Conclusions

We have cloned a novel leaf color control gene (*WLP3*) encoding a chloroplast ribosomal protein L18 involved in chloroplast growth and development in rice. We show that loss of *WLP3* function results in impaired chloroplast development in rice and that WLP3 interacts with other ribosomal subunits to co-regulate chloroplast development in rice. We are currently conducting an adetailed study to determine how mutations in *WLP3* cause leaf color abnormalities and to explore the molecular mechanisms of chloroplast ribosomal subunit interactions on chloroplast development.

## Materials and Methods

### Plant Material and Determination of Chlorophyll Content

The *wlp3* mutant was obtained by mutagenesis of japonica rice variety ZhongHua 11 (ZH11) with ethyl methanesulfonate. (Qian et al.,0.2016).Plant materials were grown under natural conditions at Zhejiang Normal University (Jinhua, Zhejiang Province) and Lingshui (Hainan Province). For hydroponic conditions, rice seedlings were placed in an artificial climate incubator and cultured in a 15 h light (33 ℃/28 ℃/23 ℃)/9 h dark (31 ℃/26 ℃/21 ℃) cycle. The second and third leaves of ZH11 and *wlp3* with the same growth patterns were collected and cut into small pieces 2–3 mm. A total of 0.1–0.2 g of the cut leaf samples were soaked in 10 mL absolute ethanol, placed in dark conditions at 4 °C, and extracted for approximately 48 h. Optical density values at three wavelengths of 665, 649, and 470 nm were measured using a spectrophotometer (DU800, BECKMAN, Fullerton, USA). Each sample was replicated in triplicate. The content of chlorophyll a (Chla), chlorophyll b (Chlb), and carotenoids (Car) was calculated using the modified formula of the Lichtenthaler method (Schoefs et al. 2001).

### Microscopic Observation and Reactive Oxygen Species Staining of *wlp3*

Leaves of WT and *wlp3* at the trifoliate stage were stored in 2.5% glutaraldehyde fixative and sent to Hangzhou Hulk Co., LTD (China) for observation of surface stomata and chloroplast ultrastructure.

Leaves from the same parts of WT and *wlp3* were cut with scissors, immersed in 0.5 mg/mL NBT and DAB solution, evacuated until the leaves were completely immersed in the solution, and stained overnight at room temperature. The NBT solution was discarded, and 95% ethanol was added to decolorize in a water bath at 80℃ until the green color of the leaves completely faded, and the results were photographed and recorded.

### Mapping Cloning and Functional Verification of ***WLP3***

The F_1_ generation plants were obtained by crossing *wlp3* with Indica 9311 and Zhefu 802 rice varieties. All individual plants exhibiting the albino phenotype in the F_2_ population were selected for DNA extraction using the CTAB method. The polymorphic markers between ZH11 and 9311 were screened using SSR and indel markers. The PCR products were separated by 4% agarose gel electrophoresis. WT and *wlp3* genomic DNA was amplified and sent to Hangzhou Tsingke Biotechnology Co., Ltd(China) for sequencing. Primer sequences are listed in Table S2. To verify the function of *WLP3*, a WLP3 complementary and GUS vectors were constructed. The *WLP3* coding region was amplified using primer pairs to construct a subcellular localization vector. The upstream 700 bp promoter and the DNA of the entire *WLP3* gene were used to construct the complementary vector. The upstream 700 bp promoter was used to construct the GUS vector. The restriction endonuclease double digest vectors pCAMBIA1300 and pCAMBIA1305.1 were used to ligate the target fragment using the homologous recombination kit Clontech In-Fusion @PCR (TaKaRa, Japan) and the enzyme cut vector. The ligation system was performed as follows: the concentration ratio of fragment to vector was 3: 1, 1 μL 5 × In-fusion HD Enzyme Premix, ddH_2_O was added to the volume of 5 μL. The constructed vector was transferred into *Escherichia coli* DH5α cells. The cultured single clones were selected and sent to Hangzhou Tsingke Biological Co., Ltd (China) for sequencing. The bacterial fluid with the correct sequence was stored and the plasmid was extracted. The extracted pCAMBIA1300-*WLP3* and pCAMBIA1305.1-*WLP3* vectors were introduced into ZH11 by Agrobacterium-mediated transformation. Transgenic results were monitored using a standard GUS staining assay. Primer sequences are listed in Table S3.

### Bioinformatics Analysis of WLP3

A predicted full-length *WLP3* protein sequence of 170 amino acids was obtained from Gramene (http://www.gramene.org/). The sequences used in the phylogenetic analysis were obtained by BLASTP searches using the *WLP3* protein sequence as a query on the National Center for Biotechnology Information database (NCBI, http://www.ncbi.nlm.nih.gov/). Full-length amino acid sequences were aligned using the DNAMAN program. Adjacency-joining trees were constructed using the bootstrap method with 1, 000 replicates using MEGA version 7.0 software. Evolutionary distances were calculated using the Poisson correction method and reflected the number of amino acid substitutions. All positions with blank and/or missing data were removed.Signal peptide prediction website useing Singalp complete (http://www.cbs.dtu.dk/services/SignalP/). Protein structure prediction using SWISS-MODEL (https://swissmodel.expasy.org/).Domain analysis was performed by SMART (http://smart.embl-heidelberg.de/smart/set_mode.cgi?NORM AL = 1). Co-expression Analysis The Rice FREND database was used for co-expression analysis of WLP3 (http://ricefrend. DNA. Affrc. Go. Jp/singleguide- gene. HTML).

### Subcellular Localization of WLP3 and Interacting Proteins

To identify the subcellular localization of *WLP3* and interacting proteins, the full-length gene coding sequences without stop codons were amplified. The resulting fragment was inserted into the GFP vector PYBA1132-GFP. The constructed vector was transformed into tobacco (Nicotiana benthamiana) and the transformed tobacco leaf cells were observed using a Zeiss LSM700 laser scanning confocal microscope (Germany). Primer sequences are listed in Table S3 and Table S9.

### RNA Extraction and Quantitative Real-Time PCR

Total RNA was extracted from rice plants using the AXYGEN kit and was reverse transcribed into cDNA using the TOYOBO (Japan) kit. The expression levels of genes related to chlorophyll synthesis and chloroplast development in ZH11 and *wlp3* were detected by qRT-PCR, using rice *OsActin* as an internal reference gene. Primer sequences are listed in Table S4-6. Four biological replicates were performed for each sample. The qRT-PCR reaction system was as follows: 1μL cDNA (concentration of 500 ng/μL), 1μL upstream and downstream primers (concentration of 10 μmol/L), and 5μL 2 × SYBR Mix. The reaction program was as follows: 95 ℃ for 30 s, 95 ℃ for 5 s, 55 ℃ for 10 s, 72 ℃ for 5 s and 55 cycles. *OsActin* was used as an internal reference gene.The quantitative results were analyzed using the 2^−△△CT^ method (Livak et al.,0.2001). The data were analyzed for significant differences using Excel and SPSS 21.0 software. Significant differences between experimental data were compared using a t-test with *P* < 0.05 indicating a significant difference.

### Abiotic Stress Treatment

After soaking and germination, seeds were transferred to 96-well PCR plates. The plate bottoms were removed and seeds were grown hydroponically in IRRI nutrient solution in an artificial climate incubator. Drought, salt, or cold treatments were applied for 3 days by treating 3-week-old plants with 20% (w/v) PEG 6000, 150 mmol L^−1^ sodium chloride (NaCl), respectively. The corresponding gene expression levels were detected by qRT-PCR. All experiments were performed in triplicate.

### Yeast Two-Hybrid Assay

*WLP3* full-length coding region was cloned into the pGBKT7 vector. Meanwhile, the full-length coding sequences encoding RPL4, RPL5, RPL9, RPS6, WGL2, and ASL2 were cloned into the pGADT7 vector. Primer sequences are listed in Table S7. The recombinant plasmids were used to transform yeast strain AH109 and the recombinant plasmids were used in SD/-Leu/-Trp and SD. Progeny were selected on /-Leu/-Trp/-His/-Ade plates.

### Bimolecular Fluorescence Complementary Detection

For bimolecular fluorescence complementation (BiFC) detection, the coding sequences of *WLP3* and RPL4, RPL5, RPL9, RPS6, WGL2, and ASL2 were cloned into nCFP and cCFP vectors, respectively. Primer sequences are listed in Table S8. The constructed vectors were then transferred into *Agrobacterium* EHA105 and then transiently co-expressed in tobacco leaf epidermal cells. Samples were imaged 24–48 h after co-transformation using a Zeiss LSM700 laser scanning confocal microscope.

### Supplementary Information

**Additional file 1**. **Fig S1**. Chlorophyll synthesis rates of WT and *wlp3*. **Fig S2**. Bioinformatics analysis of *WLP3*. **Fig S3**. Responses of WT and *wlp3* to drought stress. **Fig S4**. Responses of WT and *wlp3* to salt stress. **Fig S**5. *wlp3* interacts with other ribosomal subunits and interactional protein localization. **Fig S6**. Negative control of BiFC.

**Additional file 2**. **Table S1**. Genetic analysis of the *WLP3*. **Table S2**. Map and sequencing primers for *WLP3*. **Table S3**. Primer sequences for vector construction. **Table S4**. qRT-PCR primer sequences. **Table S5**. qRT-PCR primer sequences. **Table S6**. Stress-related qRT-PCR primer sequences. **Table S7**. Primer sequences of yeast two-hybrid vector. **Table S8**. Primer sequences for BiFC vector construction. **Table S9**. Primer sequences for interacting protein subcellular vector construction.

## Data Availability

All data generated or analysed during this study are included in this published article and its supplementary information files.

## Abbreviations

ABA: Abscisic Acid
BiFC: Bimolecular Fluorescence
Cdna: Complementary DNA
CTAB: Cetytrimethylammonium bromide
CFP: Cyan fluorescent protein
COM: Complementation
DAB: Diamino benzidine
EMS: Ethyl methyl sulfonate
GUN: Genome uncoupled
NBT: Nitrotetrazolium blue chloride
NF: Norflurazon
NEP: Nuclear.encodedpolymerase
qRT-PCR: Real-time polymerase chain reaction
PEP: Plastid.encodedpolymerase
RNA: Ribounuceleic acid
RS: Retrograde signal
InDel: Insertion-deletion
ROS: Reactive oxygen species
WLP: White leaf and panicle
