# Supplementary figures and images for: WLP3 Encodes the Ribosomal Protein L18 and Regulates Chloroplast Development in Rice
Source: Rice (N Y). 2023 Dec 13;16:59. doi: 10.1186/s12284-023-00674-9 (PMC10719208; doi:10.1186/s12284-023-00674-9)

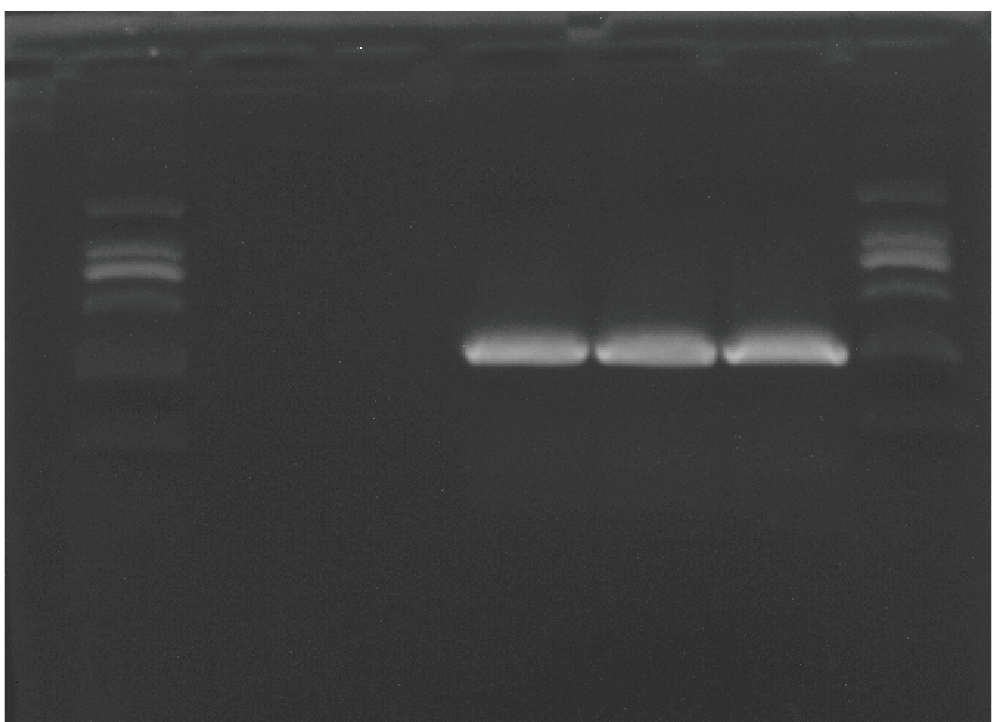


**Fig4.**(G) full uncropped Gels


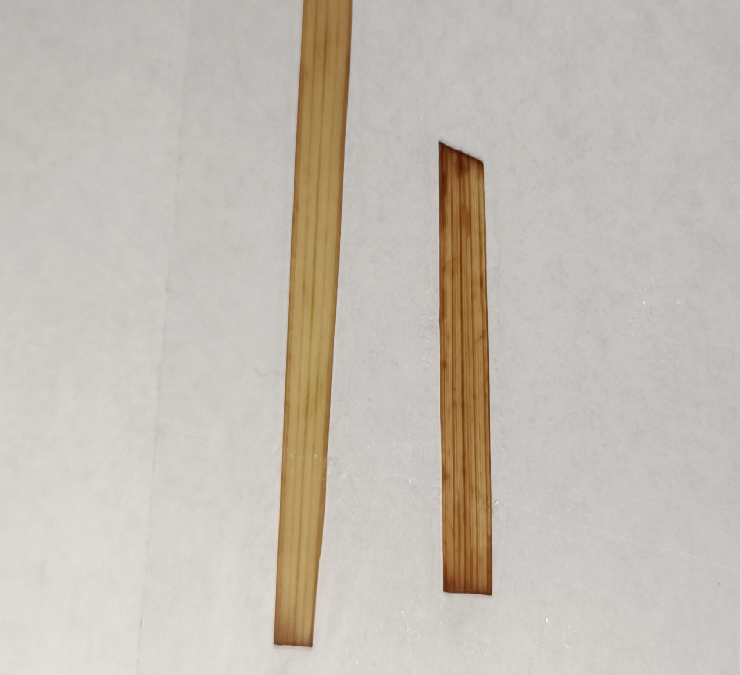

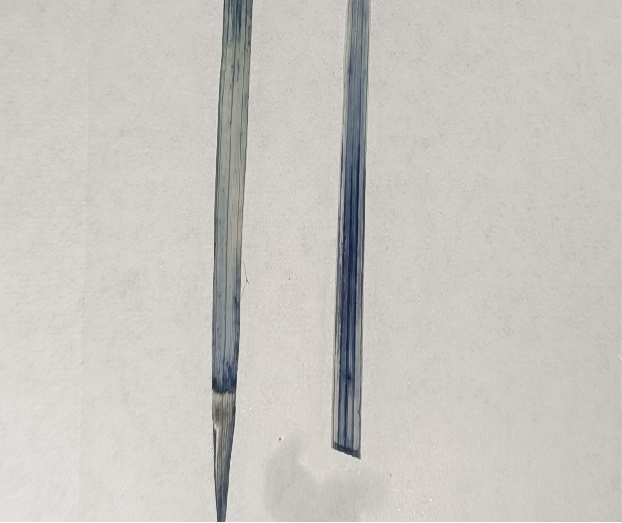


**Fig3.**(M-N) full  Blots image

Supplement: Supplementary file 1 — Additional file 1. Fig S1. Chlorophyll synthesis rates of WT and wlp3. Fig S2. Bioinformatics analysis of WLP3. Fig S3. Responses of WT and wlp3 to drought stress. Fig S4. Responses of WT and wlp3 to salt stress. Fig S5. wlp3 interacts with other ribosomal subunits and interactional protein localization. Fig S6. Negative control of BiFC. [file 12284_2023_674_MOESM1_ESM.docx]
