# Supplementary material for: WLP3 Encodes the Ribosomal Protein L18 and Regulates Chloroplast Development in Rice
Source: Rice (N Y). 2023 Dec 13;16:59. doi: 10.1186/s12284-023-00674-9 (PMC10719208; doi:10.1186/s12284-023-00674-9)
Supplement: Supplementary file 2 — Additional file 2. Table S1. Genetic analysis of the WLP3. Table S2. Map and sequencing primers for WLP3. Table S3. Primer sequences for vector construction. Table S4. qRT-PCR primer sequences. Table S5. qRT-PCR primer sequences. Table S6. Stress-related qRT-PCR primer sequences. Table S7. Primer sequences of yeast two-hybrid vector. Table S8. Primer sequences for BiFC vector construction. Table S9. Primer sequences for interacting protein subcellular vector construction. [file 12284_2023_674_MOESM2_ESM.docx]

**Supplemental information**


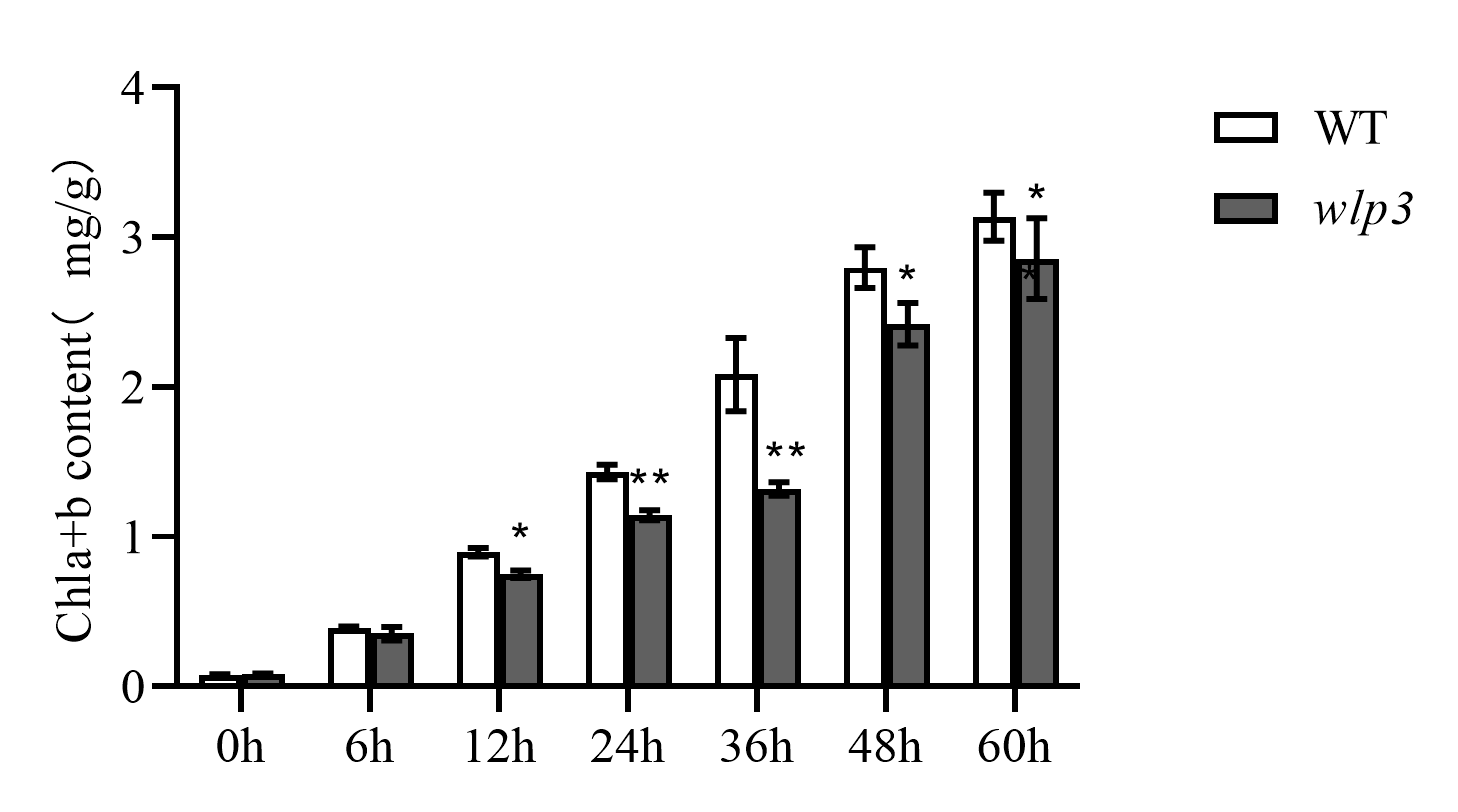


**Supplemental Fig S1.** Chlorophyll synthesis rates of WT and *wlp3*

B


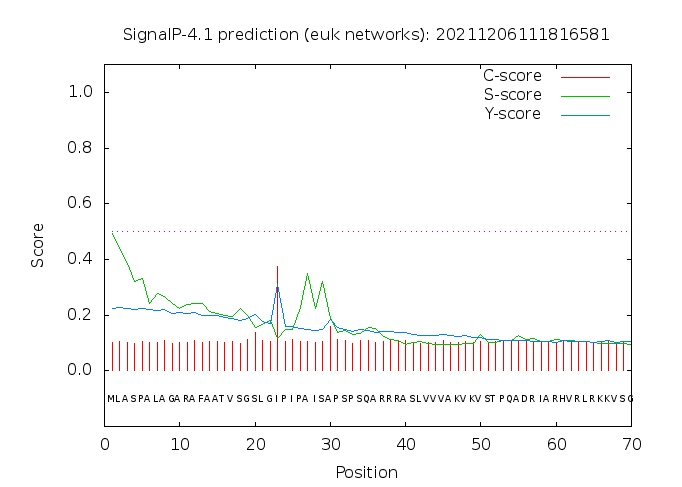

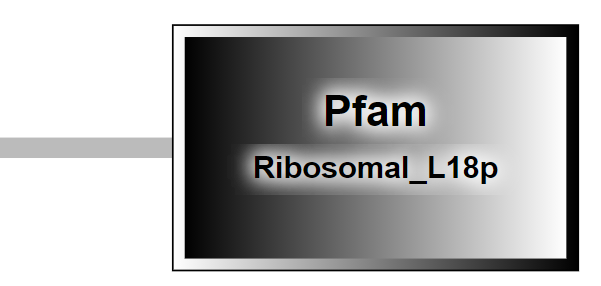

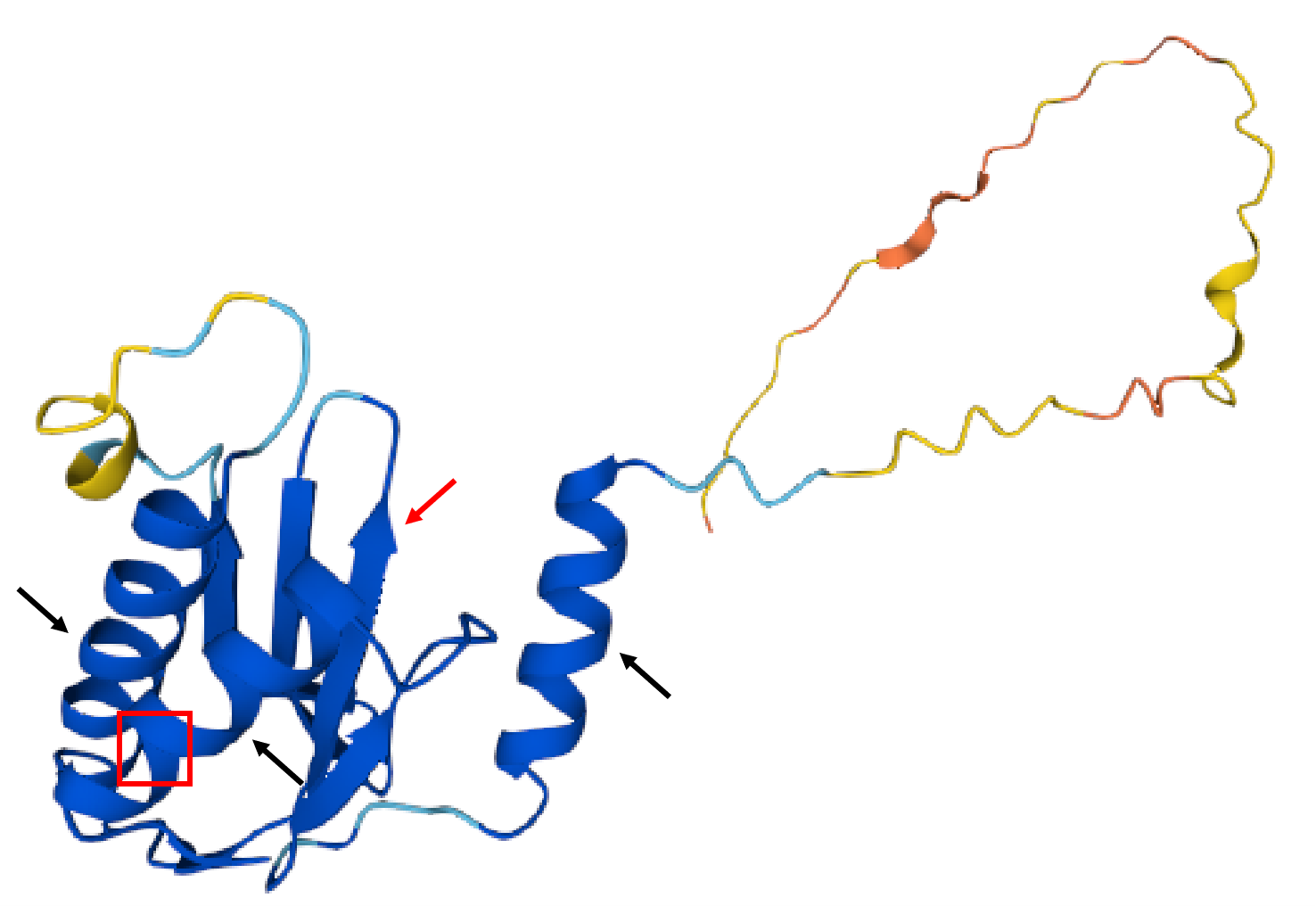

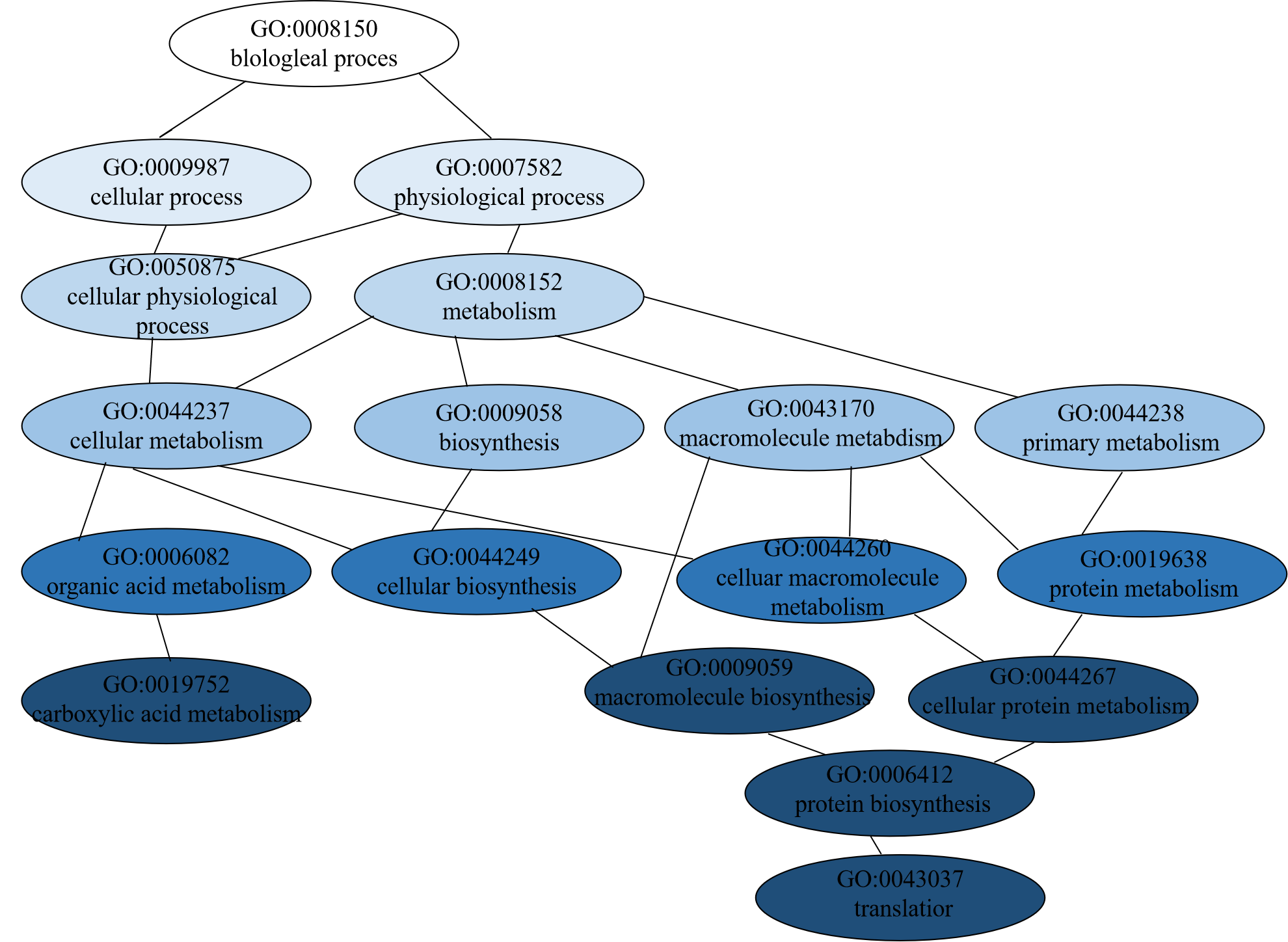


D

C

A

**Supplemental Fig S2.** Bioinformatics analysis of *WLP3*. Bioinformatics analysis of *WLP3.* (A) Signal peptide prediction of *WLP3.* (B) Pfam domain of *WLP3.* (C) 3D structure prediction of *WLP3*, red clipped Heads represent beta sheets, black arrows represent alpha helices, and red boxes represent mutation sites of *WLP3*. (D) GO enrichment analysis of *WLP3.*


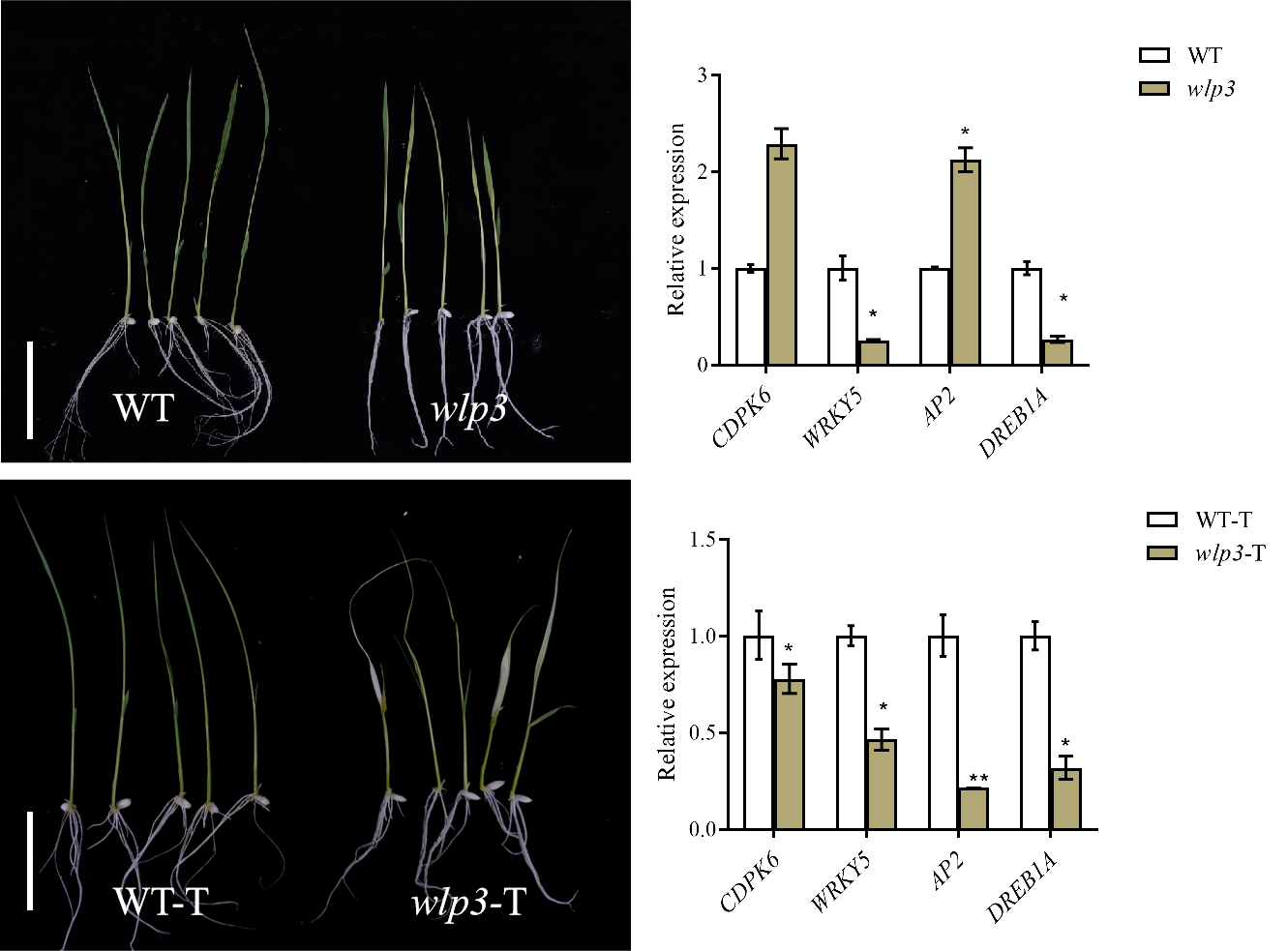


C

D

B

A

**Supplemental Fig S3.** Responses of WT and wlp3 to drought stress. (A) phenotype map of WT and *wlp3* before drought treatment ,bar=3 cm; (B) expression of genes related to drought tolerance before drought treatment of WT and *wlp3*; (C) phenotype map of WT and *wlp3* after drought treatment ,bar=3 cm; (D) Gene expression levels related to drought tolerance in WT and *wlp3* after drought treatment.


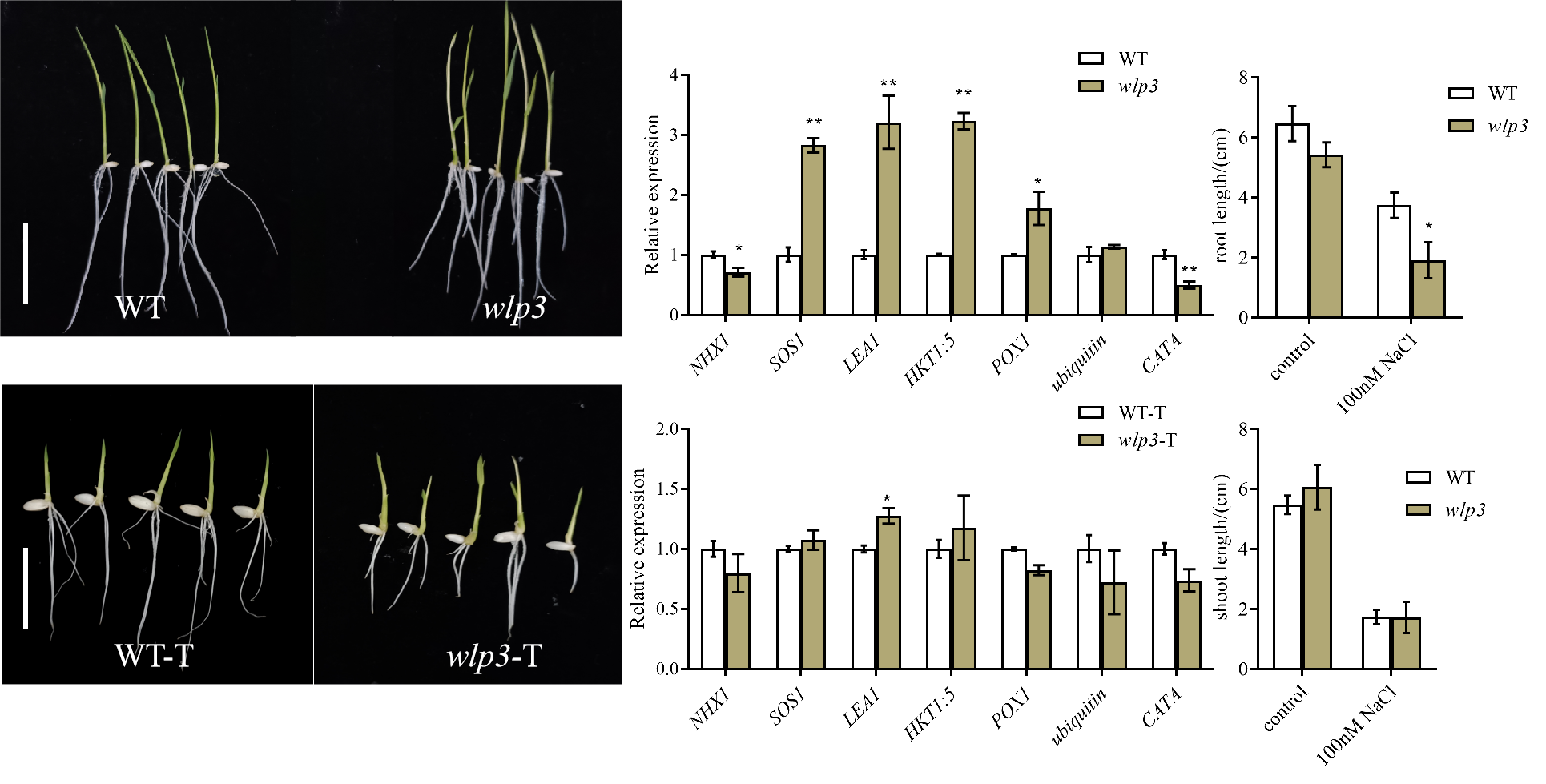


F

B

E

D

C

A

**Supplemental Fig S4.** Responses of WT and *wlp3* to salt stress. (A) Phenotypic maps of WT and *wlp3* before salt treatment, bar=2 cm; (B) expression levels of genes related to salt tolerance in WT and *wlp3* before salt treatment; (D) stem length and *wlp3* of WT and *wlp3* before salt treatment Root length; (D) phenotype maps of WT and *wlp3* after salt treatment ,bar=2 cm; (E) expression of genes related to salt tolerance in WT and *wlp3* after salt treatment; (F) WT and *wlp3* after salt treatment stem length and root length.


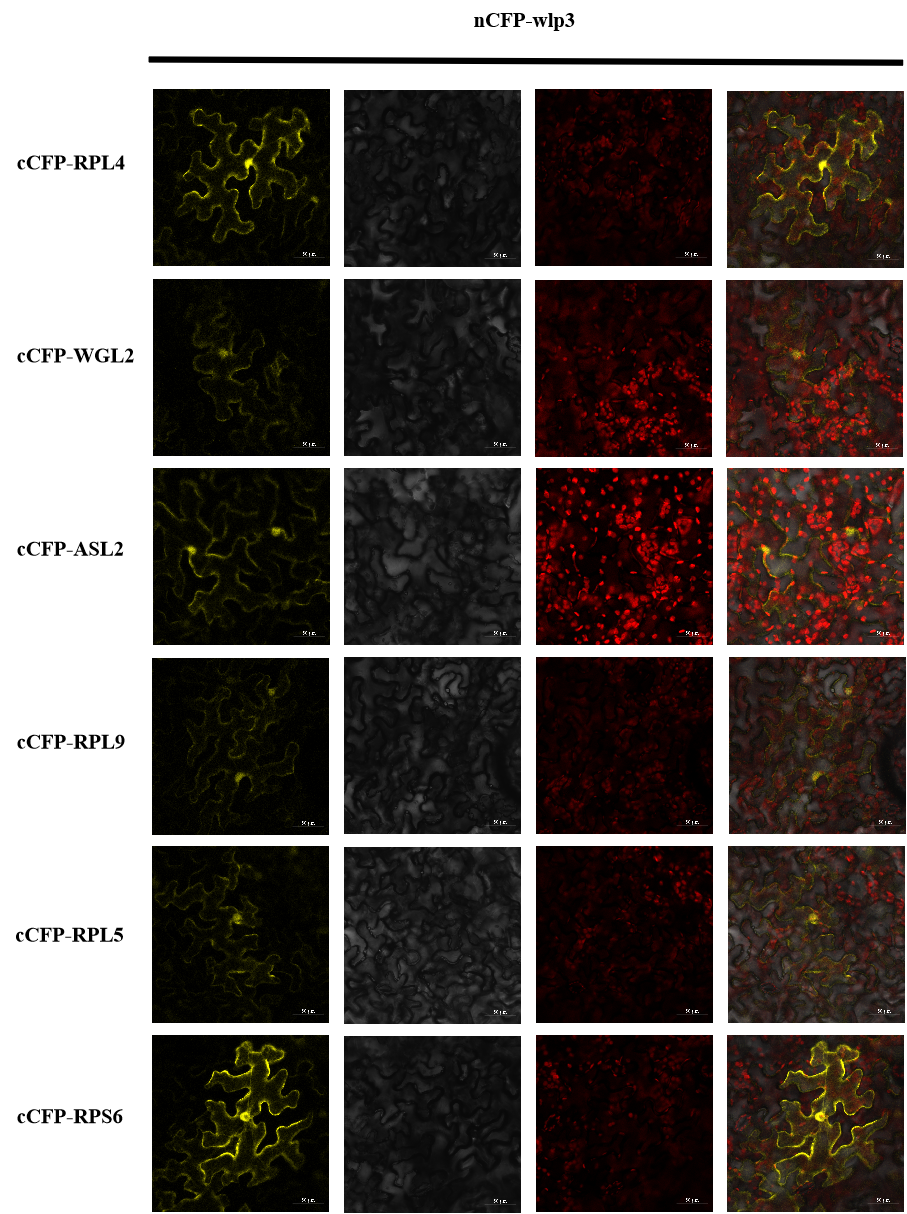

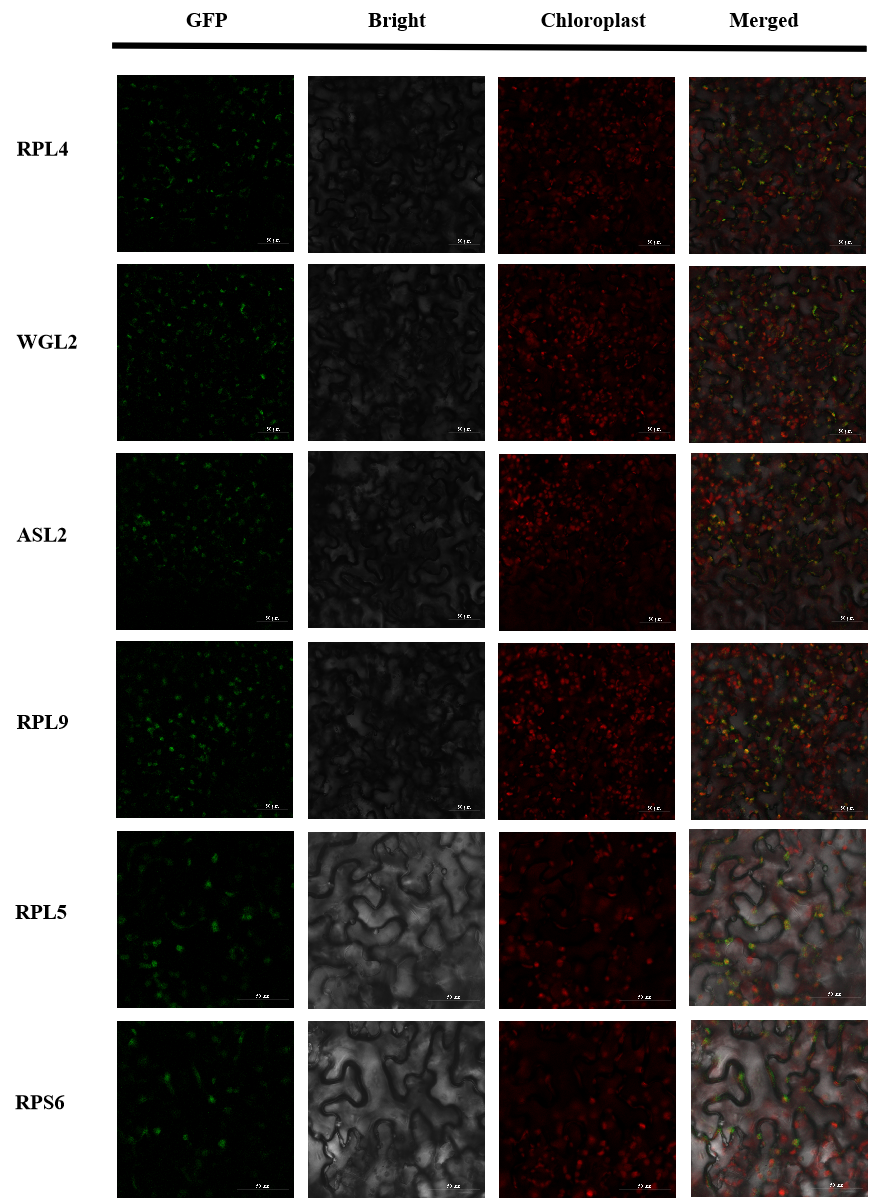

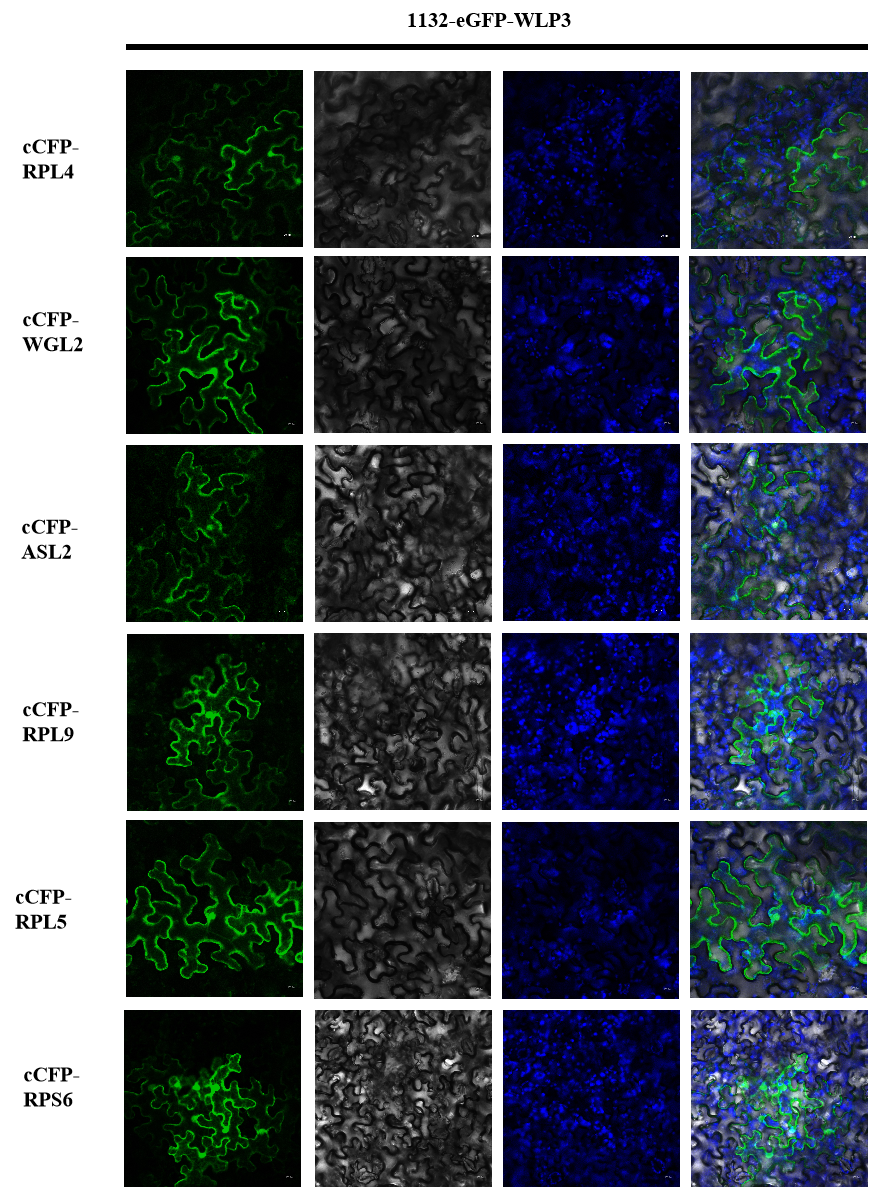


A

B

C

**Supplemental Fig S5.** *wlp3* interacts with other ribosomal subunits and interactional protein localization. (A) Verification of bimolecular fluorescence complementation of *wlp3*. (B) Subcellular localization of interacting proteins. (C) Colocalization of WLP3 with other ribosomal subunits


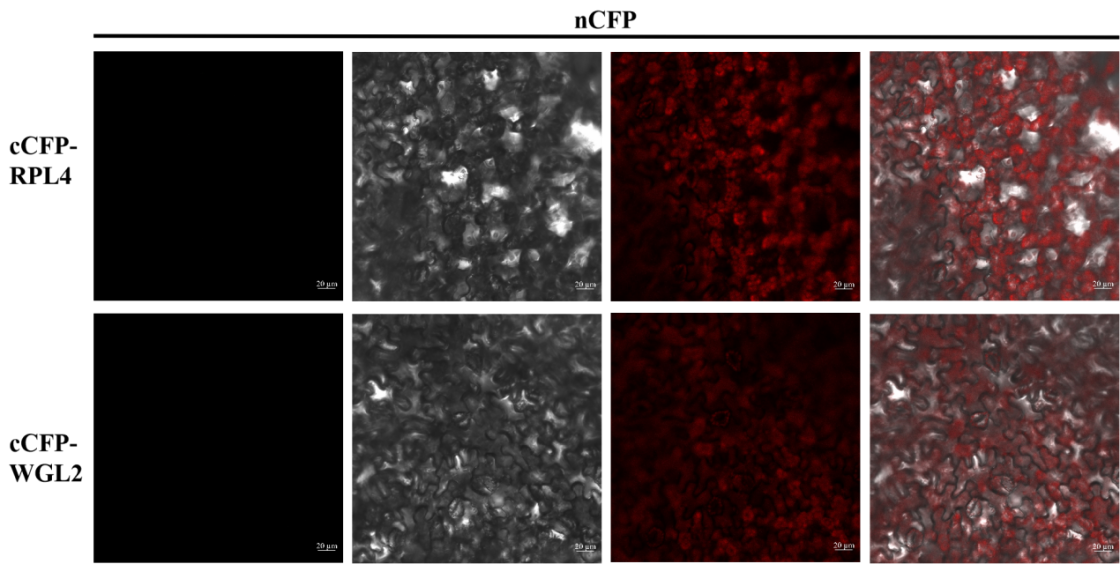

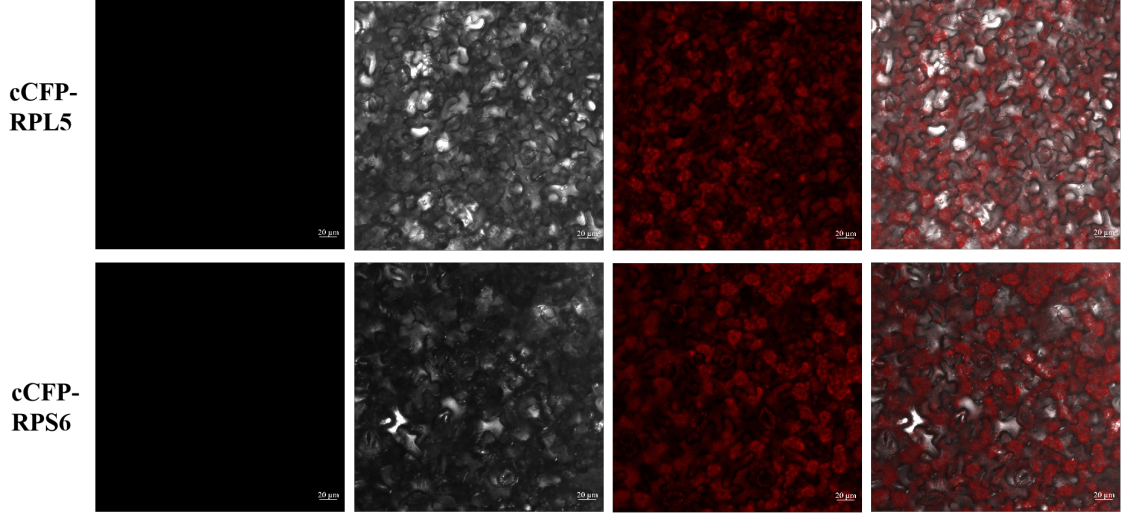

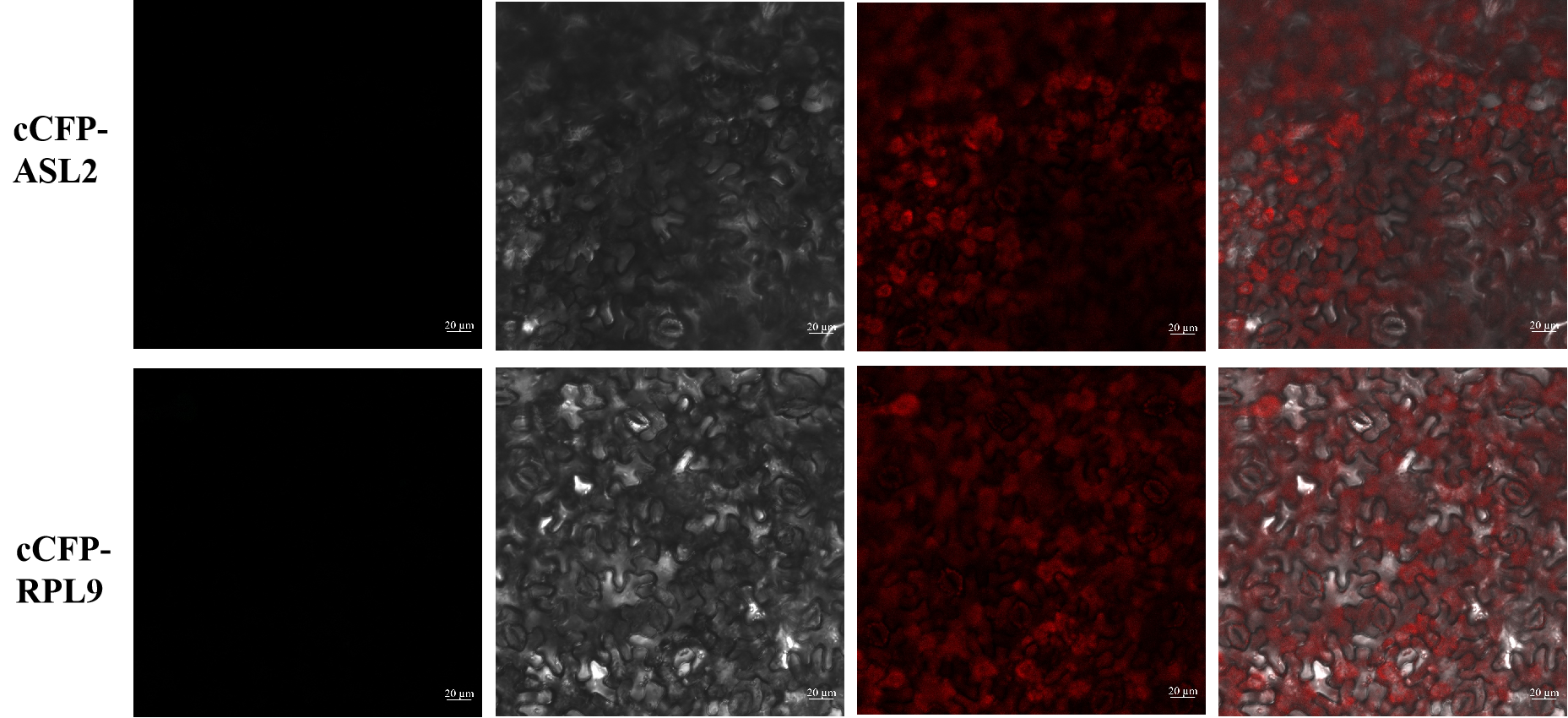

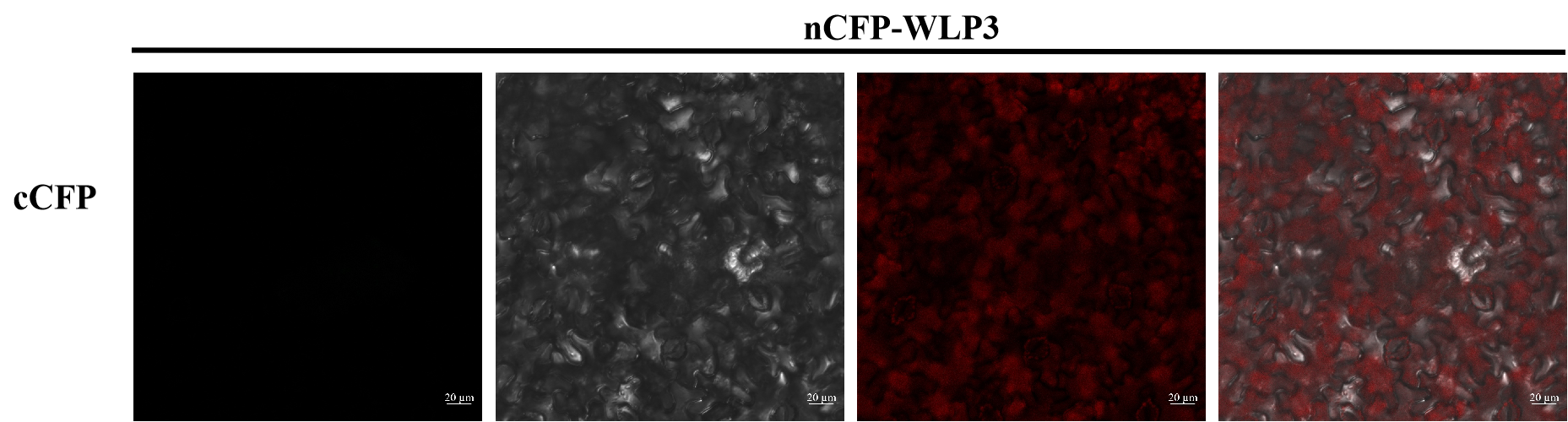


**Supplementary Fig S6.** Negative control of yeast two-hybrid and BiFC
